# Measuring evidential phenomena for complex reasoning patterns about a mass of evidence

**DOI:** 10.1007/s10506-025-09455-9

**Published:** 2025-05-03

**Authors:** Patrick Juchli, Franco Taroni, Colin Aitken

**Affiliations:** 1 School of Criminal Justice, Le Batochime, University of Lausanne, Lausanne-Dorigny, Switzerland; 2 School of Mathematics and Maxwell Institute of Mathematical Sciences, King’s Buildings, The University of Edinburgh, Edinburgh, UK

**Keywords:** Dissonant evidence, Evidence-based reasoning, Inferential interaction, Weight of evidence

## Abstract

Studies on the evidential foundations of probabilistic reasoning are extended using the notion of weight of evidence to measure evidential phenomena in the presence of a mass of evidence giving rise to complex reasoning patterns. The main results provided in this paper are methods to measure inferential interactions and dissonances among items of evidence. All measures are defined to ensure a versatile applicability in inferential tasks involving the combination of evidence and a mass of evidence. These measures enable a detailed examination of recurrent phenomena in evidence-based reasoning, such as convergence, contradiction, redundancy, and synergy. Most of these phenomena have—as far as the authors are aware—either not been formally described or any formal description proposed has been of limited use for evidence-based reasoning tasks. The present research addresses this deficit in the current understanding and treatment of these evidential phenomena. It is shown by way of examples that incorrect consideration of evidential phenomena can lead to substantial misrepresentations of the value of evidence.

## Introduction

Schum’s research on evidence-based inferences in general, and his book ‘The Evidential Foundations of Probabilistic Reasoning’ Schum (1994) in particular, are fundamental references on reasoning on combined evidence for a mass^1^ of evidence especially in judicial contexts. The present article extends and refines Schum’s approach on investigating complex reasoning patterns entailed by combined evidence and a mass of evidence. Combined evidence and a mass of evidence refer to the same concept. The latter terminology, ‘mass of evidence’, merely emphasises the large number of items of evidence that may be involved in a reasoning task.

A subjectivist or Bayesian perspective is hereby adopted (Bernardo and Smith (2000); de Finetti (2010), e.g.) − as emphasised in existing discourses on the importance of evidence for reasoning probabilistically (Schum (1994); Garrett (1998); Lindley (2006), e.g.). As the evidence changes so the associated probabilities are revised. With respect to this, the object of investigation in this paper is to extend the consideration to the different evidential phenomena emerging in arguments accommodating combined evidence and a mass of evidence. Given this context, the term ‘probabilistic reasoning’ is taken to refer to inductive, or abductive, diagnostic reasoning: that is, reasoning from evidence to the generation of propositions.

The likelihood ratio is well-documented (Good (1950); Taroni et al. (2021), e.g.) as the factor that converts prior odds in favour of a proposition to posterior odds in favour of the proposition given evidence; that is, the effect evidence has on one’s belief about the truth of a proposition (See Sect. 1.3; (Schum 1994; Taroni et al. 2004, e.g.)). This measure is considered as a variant of a confirmation measure (Crupi et al. (2007); Glass and McCartney (2015), e.g.)) and satisfies a number of necessary conditions on normative logical adequacy (see (Crupi and Tentori 2016; Taroni et al. 2021, e.g.)). Schum used the term ‘inferential force’ (exerted on some proposition) for the likelihood ratio (Schum 1994), which shall also be adopted in this paper. A slightly modified measure of inferential force is the logarithm of the likelihood ratio, known as the *weight of evidence* ( Good (1950, 1960)).

The application of the likelihood ratio to express the value of evidence in judicial litigations is well established. However, it has periodically been an object of debate in the forensic community. A more recent debate on this topic is documented in a series of publications Lund and Iyer (2017); Gittelson et al. (2018); Aitken et al. (2018). The present discussion adheres strictly to the terminology suggested in the latter publication wherein the ‘value of evidence’ refers to the likelihood ratio (of the evidence) and the ‘weight of evidence’ to the logarithm of the likelihood ratio. Both the value and the weight of evidence are regarded as logically cogent measures of the inferential force of evidence in probabilistic problem settings. Therefore, the analytical developments undertaken here rely on both the likelihood ratio and the weight of evidence.

These developments are accompanied by graphical probabilistic models in the form of Bayesian networks (Pearl (1988); Darwiche (2009); Kjaerulff and Madsen (2013), e.g.) in order to illustrate complex reasoning patterns. Bayesian networks are directed acyclic graphs constructed upon nodes and directed edges, where nodes represent random variables (propositions, intermediary reasoning steps, or observations) and edges represent conditional probabilistic relationships amongst the nodes. The details of the relationships are given in associated tables, so-called ‘node probability tables’ or NPT in short.

All the evidential phenomena addressed in this paper can be observed in the smallest possible combination of multiple items of evidence, namely the combination of two items of evidence. Schum (1994) was the first to describe most of the evidential phenomena described here. These phenomena include both harmonious and dissonant evidence along with their respective subcategories: corroboration and convergence (harmonious) as opposed to contradiction and conflict (dissonant). In addition, Schum also identified fundamental forms of inferential interactions between items of evidence, specifically, synergy, redundance and directional change.

In reasoning models that combine multiple evidence items, the relationships among items of evidence (e.g., synergy or dissonance) become central. This paper contributes a mathematical framework to describe these relational phenomena using the notion of the weight of evidence. From this framework, novel methods of analysis are derived. The utility of these methods is demonstrated by applying them to case examples.

The next three sections outline (i) generic combinations of evidence-based arguments, (ii) harmonious and dissonant evidence and (iii) inferential force and weight of evidence that form the foundation of the paper.

### Generic combinations of arguments of evidence

**Fig. 1:**
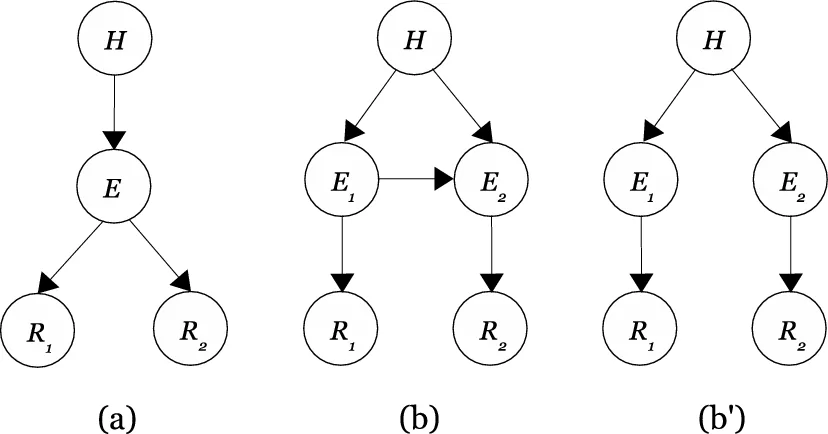
Generic types of evidence combination in the form of Bayesian networks: **a** reports $R_1$ and $R_2$ refer to the same event *E*; **b** the reports $R_1$ and $R_2$ refer to different events $E_1 \ne E_2$, which are conditionally dependent given *H*; ($\hbox {b}^{\prime }$) the reports $R_1$ and $R_2$ refer to different events $E_1 \ne E_2$ which are conditionally independent given *H*. (Figure created using Open Office Presentation)

Schum (1994) identified two generic structures for combinations of evidence. They are depicted in Fig. 1 as Bayesian networks. Each Bayesian network connects two reports $R_{1}$ and $R_{2}$, which are taken to be the evidence, about one or two events to a proposition, representing the issues of interest. Schum (1994) calls such structures ‘arguments of evidence’, which “ … serve as a reminder that inference is a human construct and that complex formal analyses of evidence are also constructed, assembled, and constituted by human actors” (Tillers 1989, p. 1253). In this sense, arguments of evidence are often built on multiple sub-arguments where each sub-argument tackles a different aspect of evidence. Such sub-arguments serve as frameworks for cascaded or hierarchical inferences (Schum and Martin 1982, e.g.). Prominent examples are the relevance of event *E* for propositions of interest *H*, or the credibility of a given report *R* regarding the occurrence of event *E*. The first type of sub-argument is called the *argument of relevance* and the second the *argument of credibility*. The distinction stems from the crucial observation − although often overlooked − that the credibility of a report about the occurrence or otherwise of an event (the argument of credibility) is different from the relevance of the event for a proposition (the argument of relevance). The argument of credibility is concerned with observer reliability and observer errors, but not the question about the relevance of the event (for discussions on observer errors in evidence-based inferences see (Good and Card 1971; Schum 1981; Thompson et al. 2003, e.g.)).

In situation (a) of Fig. 1, both reports $R_1$ and $R_2$, which may be observations or responses of a sensor, refer to the same event *E*, which, in turn, represents the relevant fact for the propositions of interest *H*. Hence, there are two arguments of credibility but only one argument of relevance. In situation (b) the two reports refer to different events, $E_1$ and $E_2$. Each of these two events is relevant for the propositions of interest. There are therefore two distinct lines of reasoning for propositions *H*. In addition, the events $E_1$ and $E_2$ are conditionally dependent given the propositions *H*, a dependence which is indicated by an edge connecting the node of $E_1$ to the node of $E_2$. Schum (1994) calls edges that directly connect two lines of argument *wefts*. The situation depicted in ($\hbox {b}^{\prime }$) is a special case of (b), in which the two events are conditionally independent given *H*, as indicated by the absence of a weft. In situation (a) $R_1$ and $R_2$ refer to what is known as the same event *E*. In situations (b) and ($\hbox {b}^{\prime }$) $R_1$ and $R_2$ refer to what is known as different events $E_1$ and $E_2$. Situation (a) is a special case of situation (b) where $E_1 = E_2$, as was shown by Schum (1994).

### Harmonious and dissonant evidence

Schum identified two distinct types for combined evidence: harmonious and dissonant evidence (Schum 1994). *Harmonious evidence* refers to situations in which two or more reports support the same proposition over the alternative. If all reports refer to the same event, then the reports form an instance of *corroborative evidence*. If, however, the reports refer to different events, then the reports are said to form an instance of *converging evidence*. Note that reports are said to *refer* to events and *support* propositions.

Unlike harmonious evidence, *dissonant evidence* refers to situations, in which two or more reports support different propositions. If the reports refer to the same event, then the reports form an instance of *contradicting evidence*. If the reports refer to different events, then the reports form an instance of *conflicting evidence*.

Thus, corroboration and contradiction inhabit the same type of argument structure (Fig. 1a). Convergence and conflict also inhabit the same type of argument structure (Fig. 1b or ($\hbox {b}^{\prime }$)). Corroboration and contradiction refer to relationships between arguments of credibility. In contrast, convergence and conflict refer to relationships between different lines of reasoning and hence arguments of relevance.

### Inferential force and weight of evidence

Schum (1994) specified that ‘evidence about some event and the actual occurrence of this event are not the same’. A report *R* of an event *E* represents evidence that *E* happened or is true^2^. *R* and *E* are not the same. Schum (1994) wrote ‘I must infer whether or not *E* actually happened or is true. Under a stipulation that all evidence is inconclusive to some degree, this inference can only be expressed in probabilistic terms’. The ‘evidence’ received is *R* the reported occurrence of an event *E*. *E* is not observed.

Consider only reports that are binary in nature. For example, this might be the outcome of a diagnostic test to detect human blood, which may be either positive or negative. Denote the two mutually exclusive outcomes of a report *R* as *r* and ${\bar{r}}$. The proposition of interest, *H*, likewise has two states; for example, the red stain at the crime scene is either human blood or some other substance. Denote these two states as *h* and ${\bar{h}}$. Background knowledge is denoted *I*. Consider an output *r* of *R* and denote the likelihood ratio

$$\begin{aligned} \frac{Pr(r \mid h, I)}{Pr(r \mid {\bar{h}}, I)} \;\; \text{ by } \;\; L_{r\mid h, I}. \end{aligned}$$

By taking the logarithm of the odds form of the Bayes’ theorem one has

1 $$\begin{aligned} \log \frac{Pr(h\mid r, I)}{Pr({\bar{h}}\mid r, I)} = \log L_{r\mid h, I} + \log \frac{Pr(h\mid I)}{Pr({\bar{h}}\mid I)}. \end{aligned}$$

where $Pr(h\mid r, I)/Pr({\bar{h}}\mid r, I)$ and $Pr(h\mid I)/Pr({\bar{h}}\mid I)$ are the posterior odds and prior odds, respectively, for proposition *h*. The likelihood ratio measures the inferential force that *r* exerts on *H*; i.e. if and to what extent *h* is supported over ${\bar{h}}$ by observing *r* and assuming that *I* holds.

Good calls the logarithm of the likelihood ratio the ‘weight of evidence’ (Good (1950, 1983), e.g.). Denote the weight of evidence, $\log L_{r\mid h, I}$, as $W_{r\mid h, I}$. *W* expresses the weight that evidence *r* provides in favour of *h* as compared to ${\bar{h}}$, given *I*. The weight takes a positive value if *r* supports the proposition in the numerator (here *h*) and negative otherwise, i.e., it supports the proposition in the denominator (here ${\bar{h}}$). The weight takes the value zero if *r* supports both propositions equally. The base of the logarithm is immaterial for the present discussion as it is the relative values of the numerator and denominator that are of concern; a base of 10 is assumed in the numerical examples. Table 6 in Appendix A outlines the extent of evidential support associated with the different likelihood ratio and weight of evidence values. For instance, the probability of an eyewitness reporting a burglary, given the proposition that a burglary actually occurred is deemed to be likely, say Pr(r∣h)=0.99. Conversely, the probability of the same eyewitness reporting a burglary when no such event occurred is considered low, i.e., $Pr(r \mid {\bar{h}}) = 0.02$. Then the likelihood ratio, or value of evidence, is given by $Pr(r \mid h)/Pr(r \mid {\bar{h}}) = 0.99/0.02 = 49.5$ which corresponds to a weight of evidence of $\log (49.5) \approx 1.7$. Following the verbal scale employed in Table 6, this corresponds to moderate evidential support for the proposition that a burglary took place.

The joint weight of evidence of *n* reports conditionally independent given *H* (as in Fig. 1 ($\hbox {b}^{\prime }$)) is the sum of the weights of evidence for each report $r_i, i\in \{1,2,...,n\}$

2 $$\begin{aligned} W_{r_1, r_2, \ldots , r_n\mid h, I} = \sum ^{n}_{i = 1}W_{r_i\mid h, I}. \end{aligned}$$

If, however, reports are not conditionally independent given *H* then

3 $$\begin{aligned} W_{r_1, r_2,\ldots , r_n\mid h, I} = W_{r_1\mid h, I} + \sum ^{n}_{i = 2}W_{r_i \mid h, r_1,\ldots , r_{i-1}, I}, \end{aligned}$$

which is a consequence of the *product rule of probability*. Using the current framework, the weight *W* a reasoner assigns to the second report $r_2$ given the first report $r_1$, the propositions *H*, and background knowledge *I*, differs from the weight assigned if $r_1$ were known. This is expressed as $W_{r_2 \mid r_1, h, I} \ne W_{r_2 \mid h, I}$. Conversely, if the weight *W* assigned to $r_2$ remains unchanged regardless of the truth of $r_1$, then the reports $r_1$ and $r_2$ are considered to be conditionally independent given *H* and *I*.^3^

The background knowledge *I* will no longer be explicitly represented, for ease of notation. However, it should be kept in mind that all probabilistic terms in this paper incorporate conditioning by *I*.

### Outline of the paper

Two measures of evidential relationships are proposed; all involve specific expressions based on the weight of evidence. These measures are discussed within the notational framework presented in Sect. 2. Section 3 presents Schum’s redundance measure, and explains how it is derived from the multiplication law of probability. Based on this measure, a more general measure for inferential interactions is established. This measure is termed the *interaction measure*.^4^ Sect. 3.5 analyses the delicate transition between two types of evidential subtleties by using the interaction measure. Section 4 introduces the *dissonance measure*. It is a measure for quantifying the amount of weight lost to dissonance in a mass of evidence. The discussion and conclusions are presented in Sect. 5.

## Framework for the analysis of masses of evidence

All generic structures for combining evidence considered here involve a divergent connection (i.e., an arrangement in which a single node has two or more direct descendants). This connection connects two or more lines of argument to a single inference stage such as an intermediary event *E* or to ultimate propositions *H*. The situation in Fig. 1a incorporates a divergent connection of the form $R_1 \leftarrow E \rightarrow R_2$ whereas (b) and ($\hbox {b}^{\prime }$) involve divergent connections of the form $E_1 \leftarrow H \rightarrow E_2$. The divergent connection is the point where different lines of argument merge and is therefore the structure that sets the stage for the emergence of different evidential phenomena for combined evidence.

**Fig. 2:**
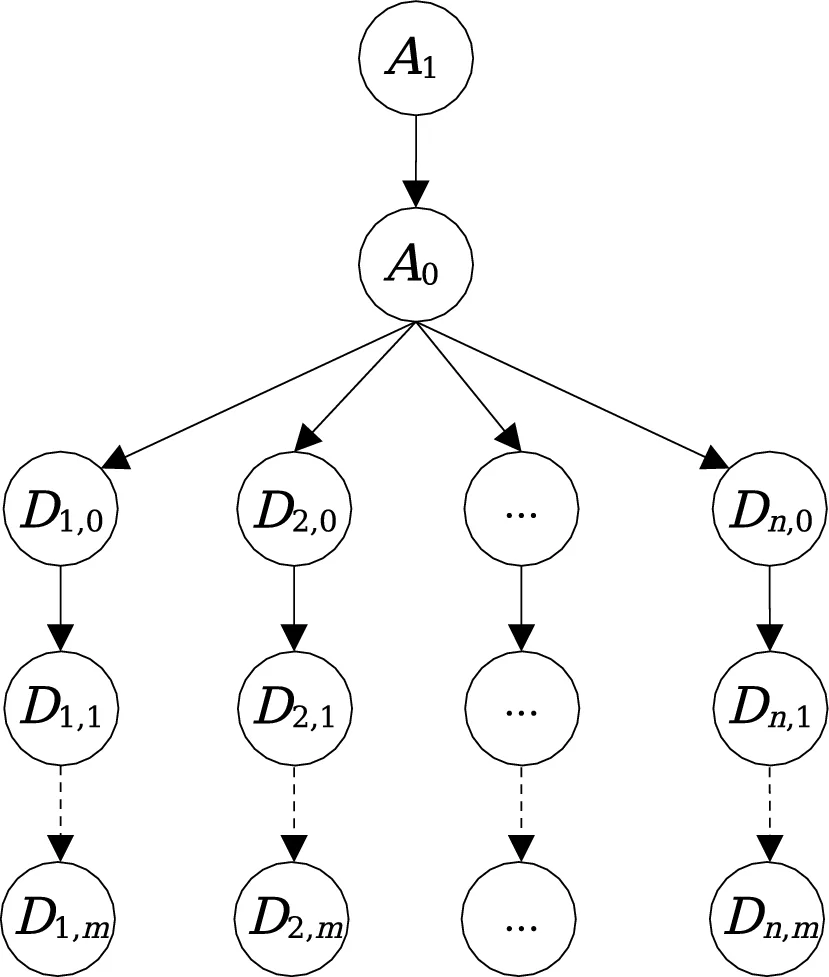
Bayesian network representing a divergent connection with multiple descendant nodes. Wefts are not shown here but may be present between any two descendant nodes. (Figure created using Open Office Presentation)

Figure 2 depicts a general representation of a divergent connection in the form of a Bayesian network. All the variables are binary. Potential wefts are omitted in Fig. 2 for increased clarity. A node that is an ancestor with respect to the divergent connection is referred to as an *ancestor* node *A*, which may be an event *E* or a proposition *H*. The ancestor node that is directly involved in the formation of a divergent connection is given index 0 and its parent index 1. For the present discussion it suffices to limit ourselves to two ancestor nodes $A_0$ and $A_1$, with $A_{1} \rightarrow A_{0}$.

A node descending from $A_0$ is referred to as a *descendant* node *D*. Descendant nodes are indexed by *i* and *j*. Index $i = \{1, 2,..., n\}$ designates the line of argument (i.e. the branch number) to which the descendant node belongs. Index $j_i$ designates the number of edges on line *i* separating a descendant node from a divergent connection. The value j=0 is given to descendant nodes directly involved in the formation of a divergent connection. Note further that $j_i = \{0, 1, 2,..., m_{i}\}$ for all $i = \{1, 2,..., n\}$ since the number of edges separating the node furthest away from the divergent connection can be different for different lines of argument .^5^

All the variables considered in this paper have only two possible responses (or states), exclusive but not necessarily exhaustive. Thus $A_{0} = \{a_{0}, {\bar{a}}_{0}\}$ and these responses could be propositions of the prosecution and defence, respectively, and $D_{i,j} = \{d_{i,j}, {\bar{d}}_{i, j}\}$ so that a single item of evidence is characterized by $d_{i,j}$ or ${\bar{d}}_{i, j}$. A set of evidence is denoted as ${\textbf{D}}$ and usually refers to the entire body of evidence. For instance, if evidence items from n=4 lines of argument, with, for example $m_{1} = 1, m_{2} = 2, m_{3} = 1, m_{4} = 0$, are considered one can refer to the body of evidence as, for example, ${\textbf{D}} = \{d_{1,0}, d_{1,1}, d_{2,0}, d_{2,1}, d_{2,2}, d_{3,0}, d_{3,1}, d_{4,0}\}$.

The body of evidence is often subdivided into mutually exclusive subsets ${\textbf{D}}_k$ and ${\textbf{D}}_{n-k}$ where the former subset contains *k* items and the latter the remaining n-k items so that ${\textbf{D}}_k \cup {\textbf{D}}_{n-k} = {\textbf{D}}$.^6^ It is assumed throughout that $Pr(D \mid a_{0})$ and $Pr(D \mid {\bar{a}}_{0})$ are both non-zero.

For the analysis of an inference structure it is often helpful to enquire on the basis of hypothetical evidence. Consider for example Fig. 1b. One can study the two reports $R_1$ and $R_2$ with regard to *H*. In contrast to this, one could also ask how a conclusion changes if, instead of $R_1$ and $R_2$, one of the events $E_1$ or $E_2$, or both were known. Such an analysis can be seen as an instance of evidential sensitivity analysis ( Jensen and Nielsen (2007)). It is driven by some hypothetical evidence claiming that $E_1$ or $E_2$ are known, as opposed to the actual reports. For the remainder of this paper, ‘evidence’ and ‘item of evidence’ are used as terms for both hypothetical evidence and actual reports.

**Example** Following the framework of Sect. 2, let the set ${\textbf{D}}$ denote the entire body of evidence, and ${\textbf{D}}_k$ and ${\textbf{D}}_{n-k}$ two arbitrary subsets of ${\textbf{D}}$. The weight provided by the body of evidence in favour of some event say, $A_0 = a_0$ (hereafter simply $a_0$ for the sake of simplicity) can be written as

$$\begin{aligned} W_{{\textbf{D}} \mid a_0} = W_{{\textbf{D}}_k \mid a_0} + W_{{\textbf{D}}_{n-k} \mid a_0}. \end{aligned}$$

By normalising this equation by the weight of the entire body of evidence $W_{{\textbf{D}} \mid a_0}$ one obtains

$$\begin{aligned} 1 = \frac{W_{{\textbf{D}}_k \mid a_0}}{W_{{\textbf{D}} \mid a_0}} + \frac{W_{{\textbf{D}}_{n-k} \mid a_0}}{W_{{\textbf{D}} \mid a_0}}, \end{aligned}$$

where the summands on the right-hand side represents fractions of weights produced by the subsets ${\textbf{D}}_k$ and ${\textbf{D}}_{n-k}$ relative to the set ${\textbf{D}}$. Assume that the weights of every single evidence in ${\textbf{D}}$ is positive (harmonious body of evidence). In such cases, an increase in *k* reduces the compound weight of ${\textbf{D}}_k$ by the same amount as the weight of ${\textbf{D}}_{n-k}$ increases. An interesting observation can be made in the extreme case where ${\textbf{D}}_{k}$ contains all the items of evidence but one, and ${\textbf{D}}_{n - k}$ incorporates the remaining *n*th item of evidence $d_{n, m_{n}}$. Moreover, assume that all lines of reasoning are independent given event $a_0$. Equation (2) suggests that the weight provided by the body of evidence in favour of $a_0$ is given by

$$\begin{aligned} \sum ^{n}_{i = 1} W_{d_{i, m_{i}} \mid a_0}&= \sum ^{n-1}_{i = 1} W_{d_{i, m_{i}} \mid a_0} + W_{d_{n, m_{n}} \mid a_0}, \end{aligned}$$

Next, consider what happens if *n* tends towards infinity. In cases where all the lines of argument provide **equal** weight of evidence in favour of $a_0$ (i.e. $W_{d_{i, j} \mid a_0} = w$, say, for all $d_{i,j} \in {\textbf{D}}$), this leads to the conclusion that additional items of evidence make decreasing contributions relative to the body of evidence

$$\begin{aligned} \underbrace{\lim _{n\rightarrow \infty ^+}\frac{\sum ^{n-1}_{i = 1}W_{d_{i,j} \mid a_0}}{\sum ^{n}_{i = 1}W_{d_{i,j} \mid a_0}}}_{ = 1} = 1- \lim _{n\rightarrow \infty ^+}\frac{W_{d_{n, j} \mid a_0}}{\sum ^{n}_{i = 1}W_{d_{i,j} \mid a_0}} = 1 - \lim _{n\rightarrow \infty ^+} \frac{w}{nw} = 1 \end{aligned}$$

and by the same token

$$\begin{aligned} \lim _{n\rightarrow \infty ^+}\frac{W_{d_{n, j} \mid a_0}}{\sum ^{n-1}_{i = 1}W_{d_{i,j} \mid a_0}} = 0. \end{aligned}$$

Identical conclusions can be made for cases where all the items of evidence provide weight of evidence in favour of ${\bar{a}}_0$ ($W_{d_{i,j_{i}} \mid a_0}<0$ for all $d_{i,j_{i}} \in {\textbf{D}}$). One can anticipate such general tendencies even in less extreme cases.

## Measuring inferential interactions

Consider two items of evidence $d_{1, 0}$ and $d_{2, 0}$, denoted $d_{1}$ and $d_{2}$ for ease of notation since given *i*, $j_{i}$ is uniquely defined, conditionally dependent given some proposition $A_0$.

Evidence $d_{1}$ is said to interact with $d_{2}$, if some knowledge about the first affects the weight of the latter in its claim about $A_0$ (one may refer to Fig. 1b with the following mapping $e_1 = d_1$ and $e_2 = d_2$ and $H = A_0$). In an effort to capture such inferential interactions, Schum (1994) introduced the so-called ‘redundance measure’ *R*^7^. In this specific situation, this redundance measure, $R_{d_2\mid d_1}$, with $W_{d_{2}\mid a_0} \ne 0$, is given by

4 $$\begin{aligned} R_{d_2\mid d_1}= & \frac{W_{d_2\mid a_0} - W_{d_2\mid a_0, d_1}}{W_{d_2\mid a_0}} = 1-\frac{W_{d_2\mid a_0, d_1}}{W_{d_2\mid a_0}}. \end{aligned}$$

Equation (4) quantifies the difference between the weight of evidence in favour of $a_0$ provided by $d_2$ alone and the weight of evidence in favour of $a_0$ provided by $d_2$ knowing that $d_1$ occurred relative to $W_{d_2\mid a_0}$. Schum showed that different values of $R_{d_2\mid d_1}$ indicate different types of inferential interactions between the events $d_1$ and $d_2$ given $A_0$ (see Appendix B). Note that $R_{d_2\mid d_1}$ measures redundancy only for the value range $R_{d_2\mid d_1} = (0, 1]$, when $0< W_{d_{2} \mid a_0, d_{1}} < W_{d_{2} \mid a_0}$ or $0> W_{d_{2} \mid a_0, d_{1}} > W_{d_{2} \mid a_0}$. For values other than these, the measure identifies other types of inferential interactions such as *synergy* or *directional change*. The generalised measure developed here will be called *interaction measure* to clarify that it measures all types of interactions and not only redundancy.

**Example.** Consider a case where a brawl between two people left one of them dead, while the other person fled the scene. A person of interest (PoI) matching the description given by several eyewitnesses is apprehended shortly after. The PoI’s clothes are confiscated and examined for blood stains. Blood stains were found on the shoes and on the sleeves of the jacket. The propositions entertained are ‘the PoI hit the victim’ ($a_0$) and ‘an unknown person, unrelated to the PoI hit the victim’ (${\bar{a}}_0$). Let $d_{1,0}$ and $d_{2,0}$ represent the DNA typing results or ‘sensor results’ (Sect. 1.1) obtained from the stains on the shoes and the jacket. Assume that the following weight values were assigned $W_{d_1\mid a_0} = W_{d_2 \mid a_0} = 1.70$ and $W_{d_2 \mid a_0, d_1} = 0.10$. The redundancy value is then given by $R_{d_2 \mid d_1} = 1 - 0.10/1.70 \approx 0.94$, which indicates that $d_2$ is redundant to with respect to $d_1$ in that $d_2$ loses 94% of its weight when $d_1$ is known (see Appendix B for the interpretation of the *R* values).

### Quantifying inferential interactions

Despite its intuitive appeal, (4) appears to be an *ad hoc* solution for the identification of inferential interactions. The present investigation suggests that this measure is in essence a normalised form of the product rule of probability. As stipulated by (2), the weight that the evidential items $d_1$ and $d_2$ provide in favour of $a_0$ can be written as

$$\begin{aligned} W_{d_1, d_2 \mid a_0} = W_{d_1\mid a_0, d_2} + W_{d_2\mid a_0} = W_{d_2\mid a_0, d_1} + W_{d_1 \mid a_0}, \end{aligned}$$

or

$$\begin{aligned} W_{d_2\mid a_0} - W_{d_2\mid a_0, d_1} = W_{d_1 \mid a_0} - W_{d_1\mid a_0, d_2}. \end{aligned}$$

Stated more succinctly, one has

5 $$\begin{aligned} W_{d_{1} \mid a_0} - W_{d_{1} \mid a_0, d_{2}} = W_{d_2\mid a_0} - W_{d_2 \mid a_0, d_1} = W_{d_1 \mid a_0} + W_{d_2\mid a_0} - W_{d_1, d_2 \mid a_0}. \end{aligned}$$

One can interpret the sum $W_{d_1 \mid a_0} + W_{d_2\mid a_0}$ as the weight provided by some ‘straw model’ *M* 8 where all the items are independent given $A_0$ and therefore devoid of any direct inferential interaction (i.e., *M* is a model obtained by removing all wefts). Let $W_{d_1, d_2 \mid M,a_0}$ denote the weight produced by the straw model in contrast to $W_{d_1, d_2 \mid a_0}$. Equation (5) can be rewritten as

6 $$\begin{aligned} W_{d_2\mid a_0} - W_{d_2 \mid a_0, d_1} = W_{d_1, d_2 \mid M,a_0} - W_{d_1, d_2 \mid a_0}. \end{aligned}$$

Any deviation of $W_{d_1, d_2 \mid a_0}$ from $W_{d_1, d_2 \mid M,a_0}$ implies the presence of a direct inferential interaction between the items. Equation (6) captures this deviation by computing the difference between $W_{d_1, d_2 \mid a_0}$ and $W_{d_1, d_2 \mid M,a_0}$. Table 8 in Appendix C outlines how the weights of $d_1$ and $d_2$ relate to a given type of inferential interaction.

**Example.** The previous example proposed the weights $W_{d_1\mid a_0} = W_{d_2 \mid a_0} = 1.70$ and $W_{d_2 \mid a_0, d_1} = 0.10$. Thus, $W_{d_2 \mid a_0} - W_{d_2 \mid a_0, d_1} = 1.70 - 0.10 = 1.60$. Following (6) one obtains the same result when computing $W_{d_1, d_2\mid M, a_0} - W_{d_1, d_2\mid a_0}$. Using the straw model *M* gives $W_{d_1, d_2\mid M, a_0} = 1.70 + 1.70 = 3.40$ and in turn $W_{d_1, d_2\mid a_0} = 1.70 + 0.10 = 1.80$. As a result, $W_{d_1, d_2\mid M, a_0} - W_{d_1, d_2\mid a_0} = 3.40 - 1.80 = 1.60$ as stipulated by the equality in (6). One can interpret $W_{d_1, d_2\mid M, a_0}$ as the standard against which $W_{d_1, d_2\mid a_0}$ is measured to uncover the inferential interaction involved. This feature serves as the bases to extend the interaction analysis to more than two lines of argument.

### Generalisation to more than two lines of argument

Following the notational framework of Sect. 2 in conjunction with the reasoning structure depicted in Fig. 2, let the set ${\textbf{D}}$ denote the entire body of evidence and $W_{{\textbf{D}} \mid a_0}$ the joint weight of evidence in favour of $a_0$ provided by ${\textbf{D}}$. The body of evidence ${\textbf{D}}$ may contain any number of lines of argument in this framework. Next, let $W_{{\textbf{D}} \mid M, a_0}$ denote the weight provided by the straw model *M*. Next, the body of evidence is split into a subset of evidence items where wefts exist and a subset where wefts do not exist. For this purpose, let the subset ${\textbf{D}}_{k}$ subsume all those *k* items of evidence contained in the body of evidence that are conditionally dependent on at least one other item of evidence given the event $A_0$. In turn, let the subset ${\textbf{D}}_{n-k}$ represent all the items that are conditionally independent given event $A_0$ so that $W_{{\textbf{D}}_{n-k} \mid M, a_0} = W_{{\textbf{D}}_{n-k} \mid a_0}$. The general form of (6) becomes

7 $$\begin{aligned} W_{{\textbf{D}} \mid M, a_0} - W_{{\textbf{D}} \mid a_0} = W_{{\textbf{D}}_k \mid M, a_0} - W_{{\textbf{D}}_k \mid a_0}. \end{aligned}$$

The weight $W_{{\textbf{D}}_k \mid M, a_0}$ is computed following (2) and $W_{{\textbf{D}}_k\mid a_0}$ following (3). The measure of *inferential interaction* denoted as $IA_{\mathbf {D_k} \mid a_0}$ is defined by normalising (7) with respect to $W_{{\textbf{D}}_k \mid M, a_0}$, assumed to be non-zero, so that

8 $$\begin{aligned} IA_{\mathbf {D_k} \mid a_0} = \frac{W_{{\textbf{D}}_k \mid M, a_0} - W_{{\textbf{D}}_k \mid a_0}}{W_{{\textbf{D}}_k \mid M, a_0}} = 1 - \frac{W_{{\textbf{D}}_k \mid a_0}}{W_{{\textbf{D}}_k \mid M, a_0}}. \end{aligned}$$

It should also be pointed out that the interaction measure can only be applied to items of evidence which are conditionally dependent on a common ancestor node (for each $D_{i, j} \in {\textbf{D}}$ the conditioning on $a_0$ must hold when formulating weights or probabilities). In Bayesian network terminology this means that all the selected nodes representing the body of evidence must be d-connected to a common ancestor node. Thus, in singly connected chains of reasoning (serially connected chains) the interaction measure cannot be applied as there is no way to select multiple descendant nodes without blocking the path between at least one descendant node and the common ancestor.

### Breaking up $W_{{\textbf{D}}_k \mid a_0}$ into types of inferential interaction

Considerations on $W_{{\textbf{D}}_k\mid a_0}$ can be further refined with respect to the components of different types of inferential interactions. Let ${\textbf{D}}_k = {\textbf{D}}_{s} \cup {\textbf{D}}_{pr} \cup {\textbf{D}}_{cr} \cup {\textbf{D}}_{dc}$ where ${\textbf{D}}_{s}$ refers to the subset of *synergistic*, ${\textbf{D}}_{cr}$ to the subset of *completely redundant*, ${\textbf{D}}_{pr}$ the subset of *partially redundant* evidence, and ${\textbf{D}}_{dc}$ to the subset of evidence inducing *directional change*. This partitioning is applied to $W_{{\textbf{D}}_k \mid a_0}$:

$$\begin{aligned} W_{{\textbf{D}}_k \mid a_0} = W_{{\textbf{D}}_{s}\mid a_0} + W_{{\textbf{D}}_{cr}\mid a_0} + W_{{\textbf{D}}_{pr}\mid a_0} + W_{{\textbf{D}}_{dc}\mid a_0}. \end{aligned}$$

Thus, one can rewrite (8) as

9 $$\begin{aligned} IA_{{\textbf{D}} \mid a_0} = 1 - \frac{W_{{\textbf{D}}_{s}\mid a_0} + W_{{\textbf{D}}_{pr}\mid a_0} + W_{{\textbf{D}}_{cr}\mid a_0} + W_{{\textbf{D}}_{dc}\mid a_0}}{W_{{\textbf{D}}_k \mid M, a_0}}, \end{aligned}$$

where, also, $W_{{\textbf{D}}_k \mid M, a_0} = W_{{\textbf{D}}_{s}\mid M, a_0} + W_{{\textbf{D}}_{pr} \mid M, a_0} + W_{{\textbf{D}}_{cr}\mid M, a_0} + W_{{\textbf{D}}_{dc} \mid M, a_0} \ne 0$. The measure $IA_{{\textbf{D}}\mid a_0}$ can computed for any particular subset and the result used to examine the interaction value for each type of inferential interaction separately. For example, if the interest is in the value of the interaction measure for synergistic evidence, then one can compute

$$\begin{aligned} IA_{{\textbf{D}}_{s} \mid a_0} = 1 - \frac{W_{{\textbf{D}}_{s}\mid a_0}}{W_{{\textbf{D}}_{s}\mid M, a_0}}, \end{aligned}$$

and analogously for the other subsets. Table 1 outlines different interaction values for each subset of evidence. A comparison of Table 1 with Table 7 reveals that the interaction values and the values of $R_{e_2|e_1}$ are identical for each type of inferential interaction. This result suggests that the inferential interaction measure proposed here is a general form of Schum’s measure (see Sect. 3). Note that for completely redundant evidence one has $W_{{\textbf{D}}_{cr}\mid a_0} = 0$ whereas $W_{{\textbf{D}}_{cr}\mid M, a_0} \ne 0$.

**Table 1:** Types of inferential interactions in terms of weight of evidence and corresponding values for the degree of inferential interaction for cases where $W_{d_{i,\cdot } \mid a_0 } > 0, d_{i,\cdot } \in {\textbf{D}}$

Definition of inferential interaction	$IA_{\cdot \mid a_0}$
Synergy	
$W_{{\textbf{D}}_{s}\mid a_0} > W_{{\textbf{D}}_{s}\mid M, a_0}$	$IA_{{\textbf{D}}_{s} \mid a_0} < 0$
Partial redundance	
$0<W_{{\textbf{D}}_{pr}\mid a_0}<W_{{\textbf{D}}_{pr}\mid M, a_0}$	$0< IA_{{\textbf{D}}_{pr} \mid a_0} < 1$
Complete redundance	
$W_{{\textbf{D}}_{cr}\mid a_0} = 0$	$IA_{{\textbf{D}}_{cr} \mid a_0} = 1$
Directional change	
$W_{{\textbf{D}}_{dc}\mid a_0}<0$	$IA_{{\textbf{D}}_{dc} \mid a_0} > 1$

### Interpreting values of $IA_{{\textbf{D}} \mid a_0}$

Most of the time the presence of inferential interactions cannot be excluded on the basis of the interaction measure unless the values of the weights for each type of interaction are known. The interaction measure is designed in such a way that a given value quantifies the cumulative impact of the inferential interactions on the joint weight of evidence $W_{{\textbf{D}} \mid a_0}$. The informative power of the interaction measure is limited to the type of interaction with the largest value in the set of evidence analysed.

In certain cases (9) allows, nonetheless, a deduction of the presence of effects of directional change and synergy. Notably if $W_{d_{i, j} \mid a0} > 0$ for all $d_{i, j} \in {\textbf{D}}_k$, an interaction value of $IA_{{\textbf{D}} \mid a_0} > 1$ implies that

10 $$\begin{aligned} IA_{{\textbf{D}} \mid a_0}> 1 \Longleftrightarrow - W_{{\textbf{D}}_{dc}\mid a_0}&> W_{{\textbf{D}}_{s}\mid a_0} + W_{{\textbf{D}}_{pr}\mid a_0} \Longrightarrow W_{{\textbf{D}}_{dc}\mid a_0}\ne 0. \end{aligned}$$

Similarly, a value of $IA_{{\textbf{D}} \mid a_0}<0$ implies that

11 $$\begin{aligned} \begin{aligned} IA_{{\textbf{D}} \mid a_0} < 0 \Longleftrightarrow W_{{\textbf{D}}_{s}\mid a_0} > W_{{\textbf{D}}_k \mid M, a_0} -W_{{\textbf{D}}_{pr}\mid a_0} - W_{{\textbf{D}}_{dc}\mid a_0} \Longrightarrow W_{{\textbf{D}}_{s}\mid a_0}\ne 0. \end{aligned} \end{aligned}$$

These statements suggest that in cases where $IA_{{\textbf{D}} \mid a_0} > 1$ or $IA_{{\textbf{D}} \mid a_0} < 0$, a directional change or synergy respectively is present in the body of evidence. However, these results also suggest that values other than the ones associated to directional change or synergy, do not allow one to conclude the absence of any inferential interactions − including directional change and synergy themselves. The proofs for the statements (10) and (11) are given in E.1 and E.2 respectively.

### The interaction measure identifies corroborative redundance as redundance

Corroborative redundance refers to the process, in which an accumulation of weight leads to a decrease in weight for any further item of evidence in a corroborative setting (cf. Figure 1a). This process is examined with respect to the interaction measure.

**Fig. 3:**
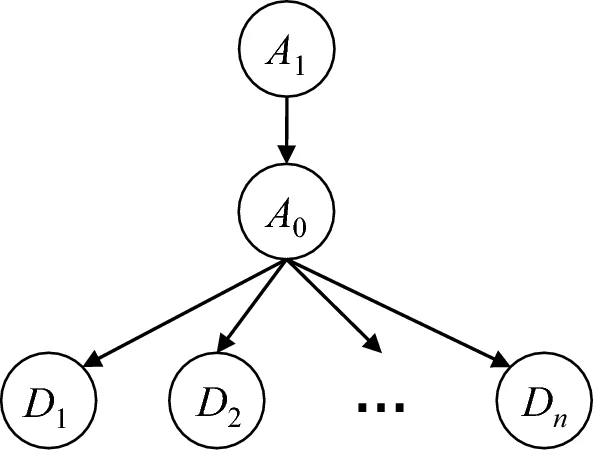
Bayesian network depicting the inference structure for corroborating evidence. For reasons of clarity the discussion focusses on one descendant node per line of argument (i.e., $${\textbf{D}} = \{D_1, D_2,..., D_n\}$$ where $$D_i = \{d_1, {\bar{d}}_1\}$$ with $$i \in \{1, 2,..., n\}$$). Notice that there are no wefts between descendant nodes $$D_i$$ and $$D_{i'}$$ where $i\neq i^{\prime }$. (Figure created using Open Office Presentation)

Let the set $\mathbf = \{d_1, d_2,..., d_n\}$ contain a series of *n* corroborative evidence items (i.e., the weights of items $d_i, i = 1, 2,.., n$ have the same sign). All items are independent given event $A_0$. Further, let the set ${\textbf{D}}$ relate to the proposition of interest $a_1$ through the intermediary event $A_0$. Figure 3 depicts the corresponding Bayesian network.

The weight of evidence provided by ${\textbf{D}}$ in favour of proposition $a_1$ is given by

12 $$\begin{aligned} W_{{\textbf{D}} \mid a_1} = \log \frac{Pr(a_0 \mid a_1) + [L_{{\textbf{D}} \mid a_0} - 1]^{-1}}{Pr(a_0 \mid {\bar{a}}_1) + [L_{{\textbf{D}} \mid a_0} - 1]^{-1}} \end{aligned}$$

where $[L_{{\textbf{D}} \mid a_0} - 1]^{-1}$ is called the *drag coefficient* Schum (1994) and $L_{{\textbf{D}} \mid a_0}$ is the local compound likelihood ratio of ${\textbf{D}}$ given $A_0$:

13 $$\begin{aligned} L_{{\textbf{D}} \mid a_0} = \prod ^n_{i=1} \frac{Pr(d_i \mid a_0)}{Pr(d_i \mid {\bar{a}}_0)}. \end{aligned}$$

The drag coefficient encapsulates the aggregate inferential force that the set of evidence ${\textbf{D}}$ exerts on $A_0$. Notice that the drag coefficient is the same in the numerator and denominator of (12). From this it follows that the aggregate weight generated by the set ${\textbf{D}}$ is bounded by the weight of $a_0$ acting on $A_1$. Stated from a graph perspective the set of nodes representing ${\textbf{D}}$ are *d*-separated from the node of $A_1$ by $A_0$.

Consider the situation in which the set ${\textbf{D}}$ provides conclusive evidence for $a_0$ to have occurred. Thus, from an algebraic point of view, the limit of the compound likelihood ratio $L_{{\textbf{D}} \mid a_0}$ (13) is infinity. In this situation the drag coefficient tends towards zero and the upper bound is $W_{a_0 \mid a_1}$.

Conversely, consider the situation in which the set ${\textbf{D}}$ provides evidence irrelevant for $a_0$ so that $L_{{\textbf{D}} \mid a_0} = 1$. The limit of the drag coefficient is infinity, which in turn produces the limiting case $W_{{\textbf{D}} \mid a_1} = 1$. This establishes that $W_{{\textbf{D}} \mid a_1} = [1, W_{a_0 \mid a_1}]$ applies for corroborating evidence.

**Fig. 4:**
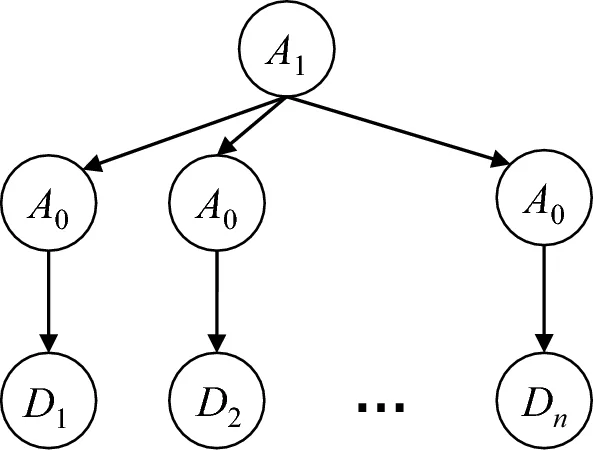
Bayesian network depicting the inference structure for corroborating evidence under the assumption that *M* holds. This corresponds to a situation of convergence, where each line of argument is independent given $a_1$. Again, for reasons of clarity the discussion focusses on one descendant node per line of argument (i.e., ${\textbf{D}} = \{D_1, D_2,..., D_n\}$ where $D_i = \{d_1, {\bar{d}}_1\}$ with $i \in \{1, 2,..., n\}$). (Figure created using Open Office Presentation.)

Next consider $W_{{\textbf{D}} \mid a_1, M}$, where following *M* one assumes that all items in ${\textbf{D}}$ are independent given $a_1$. The corresponding Bayesian network is shown in Fig. 4. In this case the weight is given by

14 $$\begin{aligned} W_{{\textbf{D}} \mid a_1, M} = \sum ^n_{i=1} \frac{Pr(a_0 \mid a_1, M) + [L_{d_i \mid a_0} - 1]^{-1}}{Pr(a_0 \mid {\bar{a}}_1, M) + [L_{d_i \mid a_0} - 1]^{-1}}. \end{aligned}$$

Note the fact that, under *M*, the weight of each evidence item $d_i$ is bounded individually by $W_{d_i \mid a_1, M} = [1, W_{a_0 \mid a_1, M}]$ for each $d_i \in {\textbf{D}}$ instead of collectively as before ($W_{{\textbf{D}} \mid a_1} = [1, W_{a_0 \mid a_1}]$). This implies that $W_{{\textbf{D}} \mid a_1, M} \ge W_{{\textbf{D}} \mid a_1} \ge 0$. Thus, if $W_{{\textbf{D}} \mid a_1, M} = W_{{\textbf{D}} \mid a_1} > 0$, then $IA_{{\textbf{D}}\mid a_1} = 0$. This situation arises when all items in ${\textbf{D}}$ have zero weight except for one and *M* does not impact the weight. Furthermore $W_{{\textbf{D}} \mid a_1, M}> W_{{\textbf{D}} \mid a_1} > 0$ applies for which $1> IA_{{\textbf{D}}\mid a_1} > 0$. As a consequence, corroborative evidence is essentially redundant in nature. This result is in agreement with the findings on corroborative redundancy by Schum and Martin (1982).

**Fig. 5:**
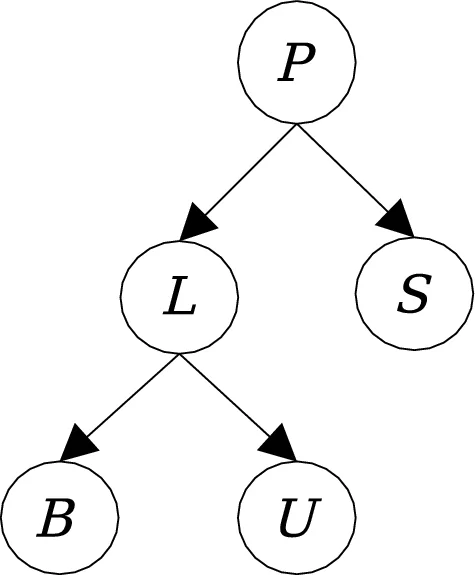
Bayesian network depicting a diagnostic model for evaluating pregnancy based on three different tests. The variable *P* denotes whether the cow is pregnant or not, *L* refers to the progesterone level, which may be detectable or not. Finally there are results from three tests: urine (*U*), blood (*B*) and scan test (*S*). In the former two, progesterone may be detectable or not and in the latter a fetus may be detectable or not. (Figure created using MS PowerPoint)

**Example** The present example is adopted from the Bayesian network discussed in Darwiche (2009) and depicted in Fig. 5. The probabilities are adjusted in the present example for clarity.

The model evaluates the pregnancy of cows after insemination based on urine (*U*), blood (*B*) and scan tests (*S*), which can be either positive (+) or negative (−). The urine and blood tests detect the progesterone level *L*, which may be detectable (*l*) or not (${\bar{l}}$). The subgraph $\{P, L, U, B\}$ corresponds to the corroboration/contradiction model and $\{L, P, S\}$ to the convergence/conflict model. The probabilities chosen in the example are purely illustrative and are not the ones used in Darwiche (2009). Table 2 shows the probability values chosen. Notice that for reasons of simplicity, the probabilities in all the descendant nodes are the same. The weights calculated based on these probability values are shown in Table 3.

**Table 2:** Probabilities used in the pregnancy test model. For simplicity, the probabilities have been chosen such that the local weight is always the same throughout the model ($W_{S=+ \mid p} = \log (0.99/0.01) \approx 2.00$)

Parent variable state	Child variable state	$Pr(\text {child state} \mid \text {parent state})$
P=p	L=l	0.99
$P={\bar{p}}$	$L={\bar{l}}$	0.01
P=p	S=+	0.99
$P={\bar{p}}$	S=+	0.01
L=l	B=+	0.99
$L={\bar{l}}$	B=+	0.01
L=l	U=+	0.99
$L={\bar{l}}$	U=+	0.01

**Table 3:** Weights of the test results

Weight of evidence	Value (rounded)
$W_{B=+, U=+ \mid P=p}$	1.99
$W_{B=+ \mid P=p}$	1.69
$W_{B=+ \mid U=+, P=p}$	0.30
$W_{U=+ \mid P=p}$	1.69
$W_{U=+ \mid B=+, P=p}$	0.30
$W_{S=+ \mid P=p}$	2.00
$W_{B=+, U=+, S=+ \mid P=p}$	3.99

The blood and urine test taken together produce a weight of 1.99 ($W_{B=+, U=+ \mid P=p}$). Each test considered in isolation produces a weight of 1.69 for each ($W_{B=+ \mid P=p}$ and $W_{U=+ \mid P=p}$). These weights can be computed using Equation (12):

$$\begin{aligned} W_{B=+, U=+ \mid P=p}&= \frac{Pr(L=l \mid P=p) + [L_{B=+ \mid l}L_{U=+ \mid l} - 1]^{-1}}{Pr(L=l \mid P={\bar{p}}) + [L_{B=+ \mid l}L_{U=+ \mid l} - 1]^{-1}} \\&= \log \frac{0.99 + [(0.99/0.01)^2 - 1]^{-1}}{0.01 + [(0.99/0.01)^2 - 1]^{-1}} \approx 1.99\\ \end{aligned}$$

and the weight for $W_{U=+ \mid P=p}$ ($W_{B=+ \mid P=p}$ is computed in the same manner)

$$\begin{aligned} W_{U=+ \mid P=p}&= \frac{Pr(L=l \mid P=p) + [L_{U=+ \mid l} - 1]^{-1}}{Pr(L=l \mid P={\bar{p}}) + [L_{U=+ \mid l} - 1]^{-1}} \\&= \log \frac{0.99 + [(0.99/0.01) - 1]^{-1}}{0.01 + [(0.99/0.01) - 1]^{-1}} \approx 1.69. \end{aligned}$$

However, if the result of one test is already known then the weight of a test is reduced to 0.30 ($W_{B=+ \mid U=+, P=p}$ or $W_{U=+ \mid B=+, P=p}$). These weights are computed using a modified form of Eq. (12) as developed in Schum (1994). The computation is shown for $W_{B=+ \mid U=+, P=p}$ (since it is essentially the same for $W_{U=+ \mid B=+, P=p}$):

$$\begin{aligned} W_{B=+ \mid U=+, P=p} = \frac{Pr(L=l \mid U=+, P=p) + [L_{B=+ \mid l} - 1]^{-1}}{Pr(L=l \mid U=+, P={\bar{p}}) + [L_{B=+ \mid l} - 1]^{-1}}, \end{aligned}$$

where

$$\begin{aligned} Pr&(L=l \mid U=+, P=p) \\&= \frac{Pr(L=l \mid P=p)Pr(U=+ \mid L=l)}{Pr(L=l \mid P=p)Pr(U=+ \mid L=l) + Pr(L={\bar{l}} \mid P=p)Pr(U=+ \mid L={\bar{l}})} \\&= \frac{0.99^2}{0.99^2 - 0.01^2} \approx = 1, \\ Pr&(L=l \mid U=+, P={\bar{p}}) \\&= \frac{Pr(L=l \mid P={\bar{p}})Pr(U=+ \mid L=l)}{Pr(L=l \mid P={\bar{p}})Pr(U=+ \mid L=l) + Pr(L={\bar{l}} \mid P={\bar{p}})Pr(U=+ \mid L={\bar{l}})} \\&= \frac{0.01\times 0.99}{0.01\times 0.99 - 0.99\times 0.01} = 0.5, \\ \end{aligned}$$

and, therefore,

$$\begin{aligned} W_{B=+ \mid U=+, P=p} = \log \frac{1 + [(0.99/0.01) - 1]^{-1}}{0.5 + [(0.99/0.01) - 1]^{-1}} \approx 0.30. \end{aligned}$$

This reduction in weight is due to corroborative reduncance. Notice that since

$$\begin{aligned} W_{U=+ \mid P=p} + W_{B=+ \mid U=+, P=p} = W_{B=+ \mid P=p} + W_{U=+ \mid B=+, P=p} = W_{B=+, U=+ \mid P=p} \end{aligned}$$

forcibly one has 1.69 + 0.30 = 1.99.

Following (8) the interaction measure yields 1-0.30/1.69≈0.82. This implies that knowledge of the result of one test reduces the weight of the second test by roughly 82%. The maximum weight that could be produced in this line of reasoning is limited by the weight of observing a detectable progesterone level directly (*L*) and is 2.00 (log(0.99/0.01)). Stated otherwise, no matter how many tests one adds to detect progesterone the latter value will never be exceeded.

Conversely, the scan test *S* is conditionally independent given pregnancy *P* and can carry its full weight of 2.00 into in the compound weight of all three tests such that 1.99+2.00=3.99 ($W_{B=+, U=+ \mid P=p} + W_{S=+ \mid P=p} = W_{B=+, U=+, S=+ \mid P=p}$).

This fact has very practical implications. If among these three tests only two can be performed (e.g, because of limitations in resources), then the reasonable choice (i.e, the choice that maximizes the compound weight of evidence) would consist in selecting either blood or urine together with the scan test. It would be unreasonable, however, to select both the blood and urine tests.

### Application of the interaction measure to forensic case examples

**Fig. 6:**
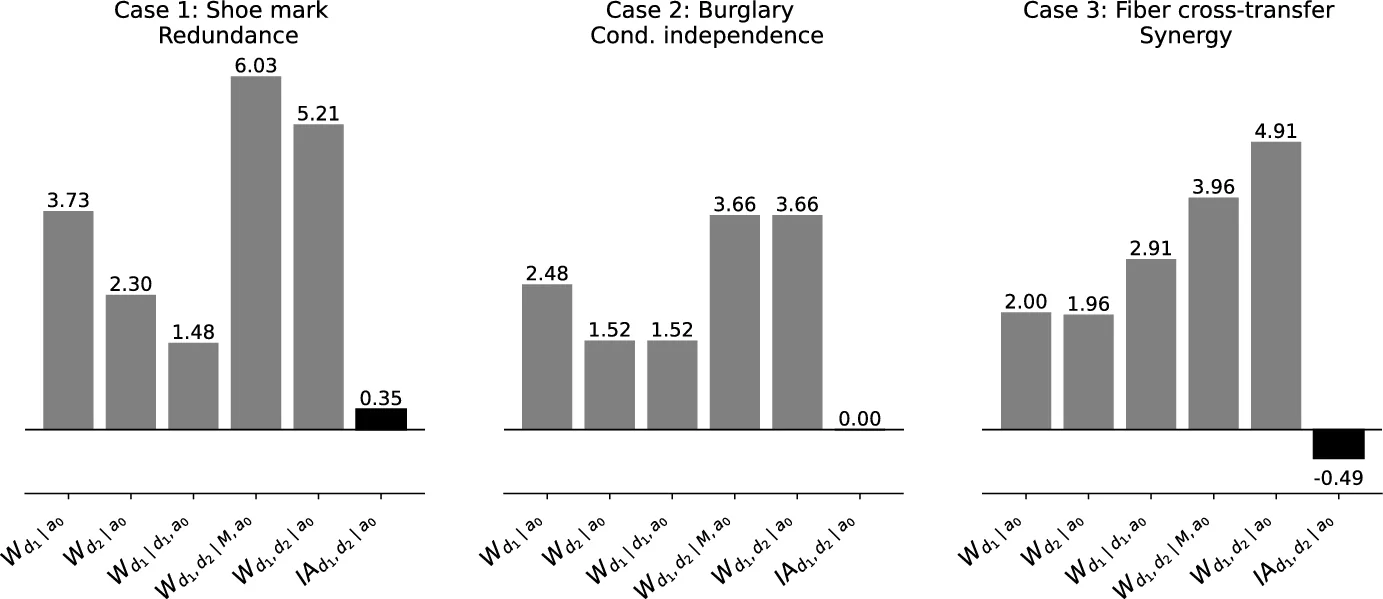
Weight values and interaction types for case examples: cases 1 and 2: Juchli et al. (2012); case 3: Taroni et al. (2020). All values are rounded to the second decimal. (Figure created using Python 3.9.12)

The interaction measure is applied to three forensic case examples. The first two are taken from real-world cases and analysed in Juchli et al. (2012), where the underlying conditional probabilities for the calculation of the weights and interaction measures may be found. The third case corresponds to a classic forensic problem and is taken from Taroni et al. (2020) (more specifically, to the values of Case 1 of the said publication). The corresponding weights and interaction values are presented in Fig. 6 for each case example.

Case 1 − ‘Shoe mark’: A woman was found dead in her bed with severe injuries caused by a third party. Several footwear marks specific to a Nike Multi Court III (size 13US) were discovered on a bathroom window, suspected as the burglar’s entry point, as well as on the upper surface of the toilet beneath the window and the bathroom floor. During the investigation the focus fell on the victim’s husband, who owned a pair of shoes of the same shoe model and size. Investigators suspected that he staged a burglary by entering through the bathroom window.

The body of evidence consists of the general pattern ($d_1$: Nike Multicourt III) and the size ($d_2$: 13US) observed in the footwear marks. The evaluation is conducted under two competing propositions: that the marks were left by the husband’s pair of shoes ($a_0$) or by a pair worn by a burglar (${\bar{a}}_0$). The calculated interaction value of 1.00-1.48/2.30≈0.35 indicates redundance, as the weight assigned to the shoe size is significantly lower when the general pattern is known ($W_{d_1 \mid d_1, a_0} = 1.48$) compared to when it is not known ($W_{d_2 \mid M, a_0} = 2.30$). If no interaction between these items had been assumed, the combined weight would have been substantially overstated as 6.03 instead of 5.21. In terms of likelihood ratio, this is roughly one million versus 160’000, leading to a 6.6 fold overstatement of inferential force.

Case 2 − ‘Burglary’: After a shop burglary, investigators found two footwear impressions behind the shop and three fingermarks on a sliding door, forming an anatomical sequence (index-middle-ring finger of a right hand) of consecutive arches (A-A-A or A-T-A). Cross-checking with staff and other circumstantial information indicated their relevance to the crime. The same day, another burglary was committed nearby, leading to the arrest of a PoI based on the victim’s description. The soles of PoI’s shoes and fingerprints both corresponded with the previously recovered marks.

The body of evidence consists of the corresponding finger ($d_1$) and footwear marks ($d_2$). Like before, the evaluation is conducted under two competing propositions: that the PoI is the burglar ($a_0$) or another unknown person is the criminal (${\bar{a}}_0$). The interaction measure yielded 1.00-1.52/1.52=0.0 indicating conditional indepence (i.e., no inferential interaction among the body of evidence) as the knowledge about the fingermark does not influence the weight of evidence assigned to the footwear impressions (the weight is 1.52 in both cases). Thus, the combined weight of evidence is identical at 3.66 which corresponds to an inferential force of 4’500 in terms of likelihood ratio.

Case 3 − ‘Fiber cross-transfer’: The fibre cross-transfer scenario refers to a situation in which direct contact occurs between two persons, two objects, or an object and a person. In such cases, a two-way or cross-transfer of fibres may take place. For instance, consider a scenario where a stolen vehicle is used in a robbery and later abandoned. A PoI is subsequently apprehended, suspected of having driven the vehicle. Upon forensic examination, a group of fibres corresponding to a garment confiscated from the PoI are discovered on the car seat. Conversely, a group of fibres corresponding to the fabric of the car seat is found on the PoI’s clothing.

The body of evidence consists of two distinct group of fibres: one group corresponds to the garment confiscated from the PoI and was detected on the car seat of the abandoned vehicle ($d_1$), while the other group corresponds to the fabric of the car seat and was found on the PoI’s clothing ($d_2$). The evaluation is again conducted under two competing propositions: that the PoI sat in the drivers seat of the vehicle ($a_0$) or that the PoI never sat in the driver’s seat (${\bar{a}}_0$). The interaction measure is calculated as 1.00-2.91/1.96≈-0.49, indicating synergy within the body of evidence. Specifically, knowledge of the first fibre group ($d_1$) significantly increases the weight of the second fibre group ($d_2$), from $W_{d_2 \mid M, a_0} = 1.96$ to $W_{d_2 \mid d_1, a_0} = 2.91$. In terms of likelihood ratio this corresponds to a ninefold increase, from approximately 90 to 800.

A well-specified reasoning model may be required to compute weights initially. That said, as demonstrated in the examples, a detailed understanding of the model’s intricacies is not necessary to employ the interaction measure, provided that the weights are available. Additionally, computing the interaction measure requires that the conditioning ancestor node of the weights (here $A_0$) has two possible responses. This requirement, however, does not extend to the descendant nodes as long as the relative sizes of the weights involved in the interaction measure can be properly evaluated. This is a a direct consequence of the definition of the inferential interaction types (see Appendix D).

During model construction, assessing whether and how conditional dependencies should be incorporated between distinct lines of reasoning (i.e., wefts) − and assigning appropriate parameters to NPTs accordingly − can be challenging. In such cases, sensitivity analyses can be useful. These analyses involve systematically modifying a model parameter to observe its impact on another quantity or exploring different scenarios to assess potential variations. Naturally, the interaction measure can also be leveraged in such analyses to clarify relationships between lines of reasoning. For instance, Juchli et al. (2012) apply sensitivity analysis to examine the interactions between various pieces of evidence, while Taroni et al. (2020) explore different scenarios to assess their impact on the interaction relationships in fiber cross-transfer settings. Consideration of sensitivity for the analyses here is beyond the scope of this paper.

## Dissonance

Two expressions involving weight can be used to measure dissonance. The first expression, is the absolute amount of weight generated by the body of evidence $\left| W_{{\textbf{D}} \mid a_0} \right|$. It can be interpreted as the weight actually expressed by the entire body of evidence considered as a whole and termed here ‘expressed weight’. Then the expressed weight of evidence is computed as

15 $$\begin{aligned} \left| W_{{\textbf{D}} \mid a_0} \right| = \left| W_{d_i \mid a_0} + \sum _{i=2}^{n} W_{d_{i} \mid a_{0}, d_{1}, \ldots , d_{i-1} }\right| \end{aligned}$$

where the index *j* is omitted for clarity.

The second expression corresponds to the sum of the absolute values of the individual weight components generated by the items in a body of evidence. That is, if the weight components are seen as a vector then this expression corresponds to its $l_1$-norm (for clarity, the subscript 1 is omitted). It can be seen as the amount of weight existing not as an actual manifestation of the weight in a body of evidence but only as a potential maximum for the body of evidence. This ‘potential weight’ is computed as

16 $$\begin{aligned} \Vert W_{{\textbf{D}} \mid a_0} \Vert&= \left| W_{d_{1} \mid a_0}\right| + \sum ^n_{i = 2}\left| W_{d_{i} \mid a_0, d_{1}, \ldots , d_{i-1}}\right| . \end{aligned}$$

where index *j* is again omitted for clarity.

By definition of the norm $\Vert \cdot \Vert$, $0 \le \Vert W_{{\textbf{D}} \mid a_0} \Vert$ holds with equality only if each weight component is zero. In contrast, if the set ${\textbf{D}}$ relates to completely dissonant evidence, then the expressed weight is zero ($\left| W_{{\textbf{D}} \mid a_0} \right| = 0$). Moreover, if the body of evidence is dissonant to some degree, then the expressed weight is smaller than the potential weight ($\left| W_{{\textbf{D}} \mid a_0} \right| < \Vert W_{{\textbf{D}} \mid a_0} \Vert$) (an example of the triangle inequality). Finally, if ${\textbf{D}}$ relates to a body of harmonious evidence, then both the potential and the expressed weights are the same ($\left| W_{{\textbf{D}} \mid a_0} \right| = \Vert W_{{\textbf{D}} \mid a_0} \Vert$). The triangle inequality of real numbers holds so that

17 $$\begin{aligned} \left| W_{{\textbf{D}} \mid a_0} \right| \le \Vert W_{{\textbf{D}} \mid a_0} \Vert . \end{aligned}$$

A quantitative comparison is obtained by formulating the amount of dissonance in the weight $W_{{\textbf{D}} \mid a_0}$ as the difference between the weight potential and the expressed weight:

18 $$\begin{aligned} W^\text {diss}_{{\textbf{D}} \mid a_0} = \Vert W_{{\textbf{D}} \mid a_0} \Vert - \left| W_{{\textbf{D}} \mid a_0} \right| . \end{aligned}$$

Equation (18) measures the dissonance as the amount of potential weight lost due to dissonance in the body of evidence.

From (17), it follows directly that $0 \le W^\text {diss}_{{\textbf{D}} \mid a_0}$. Note also that if the items provide no weight at all (i.e., $\Vert W_{{\textbf{D}} \mid a_0} \Vert = \left| W_{{\textbf{D}} \mid a_0} \right| = 0$), then the weight lost due to dissonance is zero. Table 4 summarises the relationship between values of $W^\text {diss}_{{\textbf{D}} \mid a_0}$ and the characteristics of the evidence in terms of harmony and dissonance.

**Table 4:** Different values of $W^\text {diss}_{{\textbf{D}} \mid a_0}$, its implication on the relationship between $\Vert W_{{\textbf{D}} \mid a_0} \Vert$ and $\left| W_{{\textbf{D}} \mid a_0} \right|$, and on the characteristics of the body of evidence

$W^\text {diss}_{{\textbf{D}} \mid a_0}$	$\Vert W_{{\textbf{D}} \mid a_0} \Vert$ and $\left| W_{{\textbf{D}} \mid a_0} \right|$	Characteristics
$\Vert W_{{\textbf{D}} \mid a_0} \Vert$	$\left| W_{{\textbf{D}} \mid a_0} \right| = 0$	Maximum dissonance
$$0< W^{\text {diss}}_{{\textbf{D}} \mid a_{0}} <$$$$\Vert W_{{\textbf{D}} \mid a_0} \Vert$$	$$\Vert W_{{\textbf{D}} \mid a_0} \Vert> \left| W_{{\textbf{D}} \mid a_0} \right| > 0$$	Dissonant evidence
0	$\Vert W_{{\textbf{D}} \mid a_0} \Vert = \left| W_{{\textbf{D}} \mid a_0} \right|$	Harmonious evidence

Further, let ${\textbf{D}}_k$ denote the subset of ${\textbf{D}}$ that contains the items providing positive weights, and ${\textbf{D}}_{n-k}$ negative weights so that $W_{{\textbf{D}}_{k} \mid a_0} > 0$ and $W_{{\textbf{D}}_{n - k} \mid a_0} < 0$. This reduces (18) to

19 $$\begin{aligned} W^\text {diss}_{{\textbf{D}} \mid a_0} = {\left\{ \begin{array}{ll} 2W_{{\textbf{D}}_{k} \mid a_0},& \text {for } W_{{\textbf{D}}_{k} \mid a_0}<-W_{{\textbf{D}}_{n - k} \mid a_0},\\ W_{{\textbf{D}}_{k} \mid a_0}-W_{{\textbf{D}}_{n - k} \mid a_0},& \text {for } W_{{\textbf{D}}_{k} \mid a_0} = -W_{{\textbf{D}}_{n - k} \mid a_0},\\ -2W_{{\textbf{D}}_{n - k} \mid a_0},& \text {for } W_{{\textbf{D}}_{k} \mid a_0} > -W_{{\textbf{D}}_{n - k} \mid a_0}. \end{array}\right. } \end{aligned}$$

A formal development leading to (19) is given in Appendix F.

The measures of dissonance represent at the same time measures of contradiction or conflict (see Sect. 1.2). More precisely, if the argument structure under examination corresponds to the one of corroboration and contradiction, then naturally the measure of dissonance is a measure of contradiction. If the argument structure corresponds to the convergence and conflict model, then the measure of dissonance is a measure of conflict.

### Comparing dissonances

Dissonances are best compared in relative terms rather than on their own (i.e., $W^\text {diss}_{{\textbf{D}} \mid a_0}$). It is often more informative, to examine the weight lost to dissonance $W^\text {diss}_{{\textbf{D}} \mid a_0}$ relative to the corresponding weight potential $\Vert W_{{\textbf{D}} \mid a_0} \Vert$, rather than $W^\text {diss}_{{\textbf{D}} \mid a_0}$ alone:

$$\begin{aligned} \frac{W^\text {diss}_{{\textbf{D}} \mid a_0}}{\Vert W_{{\textbf{D}} \mid a_0} \Vert } = 1 - \frac{\left| W_{{\textbf{D}} \mid a_0} \right| }{\Vert W_{{\textbf{D}} \mid a_0} \Vert }, \end{aligned}$$

where $\Vert W_{{\textbf{D}} \mid a_0} \Vert \ne 0$, $\Vert W_{{\textbf{D}} \mid a_0} \Vert \ne \infty$, and $\left| W_{{\textbf{D}} \mid a_0} \right| \ne \infty$. The ratio on the left-hand side of the equation will range from 0 (indicating minimum dissonance) to 1 (indicating maximum dissonance).

### Example: directional change is a form of dissonant evidence

As pointed out in Sect. 3, an effect of directional change entails the combined weight of evidence of two items $d_{1}$ and $d_{2}$ to be smaller than that of $d_{1}$ alone in situations where all the items provide weight in favour of, say, $a_0$ ($W_{d_{1}, d_{2} \mid a_0} < W_{d_{1} \mid a_0}$). This is due to the fact that a directional change is defined as $W_{d_{2}\mid a_0, d_{1}} < 0$ (see Appendix D). Thus, the joint weight of the items $d_{1}$ and $d_{2}$ is, by definition, smaller than the weight of these items in absolute terms ($\left| W_{d_{1} \mid a_0} +W_{d_{2}\mid a_0, d_{1}}\right| < \left| W_{d_{1} \mid a_0}\right| + \left| W_{d_{2}\mid a_0, d_{1}}\right|$) so that $0 < W^\text {diss}_{{\textbf{D}} \mid a_0}$. In other words, evidence involving directional change is necessarily dissonant to a certain degree (cases of directional change where $W_{d_{1} \mid a_0} < 0$ and $W_{d_{2}\mid a_0} < 0$ lead to the same conclusion).

### Example: propagation of dissonance along lines of reasoning

A short discussion on the weight in a single line of reasoning serves as a starting point. This discussion is then extended to clarify how dissonance propagates along multiple chains of reasoning.

**Fig. 7:**
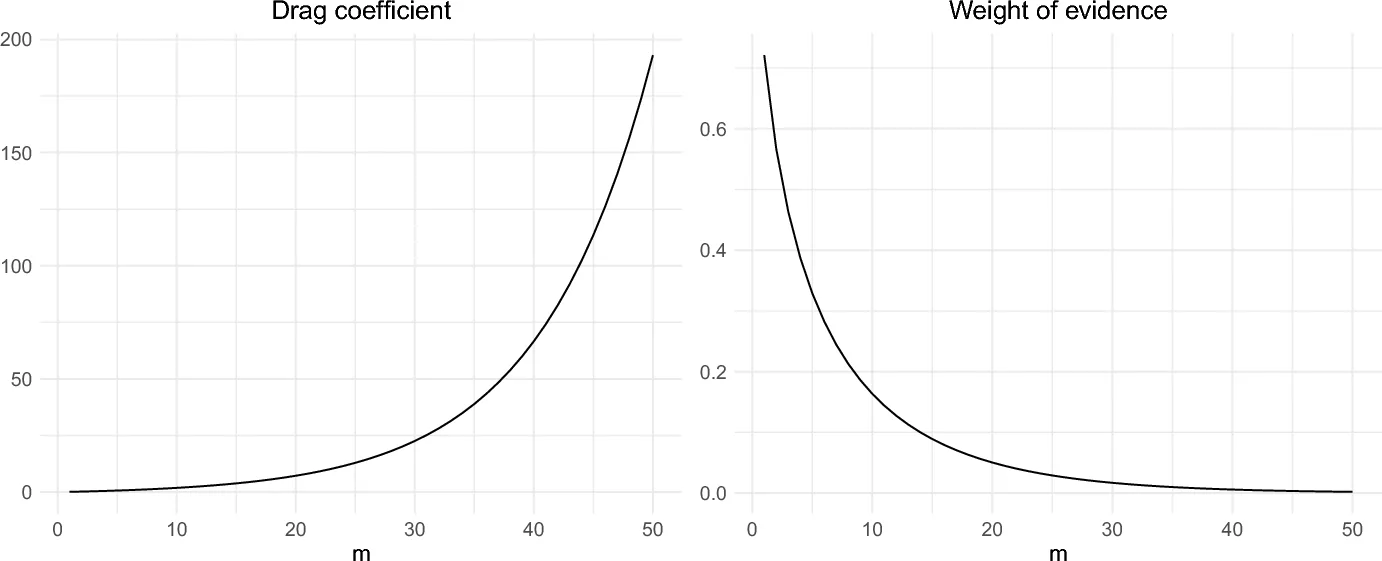
The drag coefficient (left) and the weight of evidence (right) plotted as a function of stages $m = \{1, 2,..., 50\}$ with $\alpha _j = 1.0$ and $\beta _j = 0.1$ at each stage. (Figure created using R version 4.3.1)

Take a single chain of reasoning with a total of *m* stages $A_0 \rightarrow D_1 \rightarrow D_2 \rightarrow ... \rightarrow D_m$, where $A_0 = \{a_0, {\bar{a}}_0\}$ and $D_j = \{d_j, {\bar{d}}_j\}$ with $j \in \{0, 1,..., m\}$. Let $\alpha _0 = Pr(d_0 \mid a_0)$ and $\beta _0 = Pr(d_0 \mid {\bar{a}}_0)$, and $\alpha _j = Pr(d_j \mid d_{j-1})$ and $\beta _j = Pr(d_j \mid {\bar{d}}_{j-1})$ with *j* starting at stage j=1. The weight at the *m*th stage is given by

$$\begin{aligned} W_{d_m\mid a_0} = \log \frac{ \alpha _0 + \sum ^m_{j=1}\frac{\beta _j}{\prod ^m_{j=1}(\alpha _j - \beta _j)} }{ \beta _0 + \sum ^m_{j=1}\frac{\beta _j}{\prod ^m_{j=1}(\alpha _j - \beta _j)} }, \end{aligned}$$

where $\sum ^m_{j=1}\frac{\beta _j}{\prod ^m_{j=1}(\alpha _j - \beta _j)}$ is again the drag coefficient already mentioned in Sect. 3.5. This time, however, it is developed with respect to the chain length (see Schum (1994)). Given that the drag coefficient is the same in the numerator and denominator, the fraction will tend towards unity and the weight towards zero the bigger the drag coefficient becomes relative to the probabilities $\alpha _0$ and $\beta _0$. This will happen as long as $\beta _j$ is larger than zero and $\alpha _j \ne \beta _j$ in every stage *j* with increasing *m*. Figure 7 illustrates a numerical example, where the drag coefficient and weight of evidence are plotted as a function of the number of stages *m*. It shows a situation, where $\alpha _j> \beta _j > 0$ and are constant at every stage *j*.

If a total of *n* chains of reasoning of the same length *m* are combined such that they are only connected via $A_0$ (i.e., there are no wefts between the chains), then the weight of all the chains combined is the sum of the weights produced by each chain (see (2)). In this case the dissonance is given by

$$\begin{aligned} W^\text {diss}_{{\textbf{D}} \mid a_0} = \Vert W_{{\textbf{D}} \mid a_0} \Vert - \left| W_{{\textbf{D}} \mid a_0} \right| = \sum ^n_{i=1}\left| W_{d_i \mid a_0}\right| - \left| \sum ^n_{i=1}W_{d_i \mid a_0}\right| \end{aligned}$$

(index *j* is again omitted for clarity). Assume that the dissonance is introduced at the first stage j=0 (e.g., $W_{d_{1, 0} \mid a_0} < 0$ and $W_{d_{i, 0} \mid a_0} > 0$ for all $i = \{2, 3,..., n\}$). Assume moreover that $\beta _{i, j} > 0$. In this situation, a stage by stage increase of the chain length increases also the size of the drag coefficient such that the weight in each chain tends towards zero. That is, chains having positive weights will approach zero from above and chains with negative weights will approach zero from below. This implies that the longer the chain the smaller the weights involved and the smaller the dissonance.

### Application of the dissonance measure to a case example

The example presented here is the Italian Taffio Palmi - Busetto case discussed in De March and Taroni (2020) and Taroni et al. (2024). This example utilises the model from the former to study the DNA evidence of the case.^9^ The weight and dissonance values are provided in Table 5.

Taffio Palmi was found stabbed to death in her apartment, where she lived alone. Witnesses reported that the victim regularly wore a golden necklace, which was missing from the crime scene. About a month later, Busetto was apprehended, and a broken necklace was found in her apartment. While Busetto claimed that it was a family heirloom, which had not been touched for years, the prosecution argued that it belonged to Taffio Palmi and was taken during the attack. Forensic analysis yielded amgibuous results: A first laboratory found no human cells using a presumptive test or DNA on the necklace. The analysis is repeated a few months later by a second laboratory and obtains a full DNA profile corresponding to Taffio Palmi's profile from a small quantity of DNA. The latter finding was used in the trial, where the court ultimately ruled that the necklace belonged to Taffio Palmi and was taken by Busetto during the fatal assault.

The body of evidence ${\textbf{D}}$ under consideration consists of three items of evidence. The first two items − the negative result from the presumptive test ($d_1$) and the negative DNA profile ($d_2$) − originate from the first laboratory’s analysis. The third item is the DNA profile corresponding to the victim’s genotype, produced by the second laboratory from a small quantity of DNA ($d_3$). These pieces of evidence are evaluated under two competing propositions. The prosecution’s proposition asserts that Busetto took the necklace from the victim during the attack ($A_0 = a_0$). In contrast, the defence claims that the necklace belongs to Busetto and had not been touched for years ($A_0 = {\bar{a}}_0$). The weight of the first and second items are negative and positive for the third. The expressed weight is $\left| W_{{\textbf{D}} \mid a_0} \right| = \left| -0.82 + -0.23 + 1.11 \right| = 0.06$ and the potential weight $\Vert W_{{\textbf{D}} \mid a_0} \Vert = \left| -0.82 \right| + \left| -0.23 \right| + \left| 1.11 \right| = 2.16$. Thus, the amount of weight at dissonance is $W^\text {diss}_{{\textbf{D}} \mid a_0} = \Vert W_{{\textbf{D}} \mid a_0} \Vert - \left| W_{{\textbf{D}} \mid a_0} \right| = 2.16 - 0.06 = 2.10$ and the fraction of weight lost to dissonance 97% ($W^\text {diss}_{{\textbf{D}} \mid a_0} = 1 - 0.06/2.16 \approx 0.97$), an almost total loss of weight due to dissonance. The actual weight of evidence is 0.06, indicating that the body of evidence has weak support for the prosecution’s proposition over the defence’s. In terms of likelihood ratio this corresponds to $1/10^{0.06} \approx 1.15$, a value at the lower end of the ‘weak’-category with regards to the verbal scale used in Table 6. The weight (2.10) lost to dissonance corresponds to a value of evidence of more than 120 ($10^{2.10} \approx 126$) which translates to strong evidential support (see Table 6).

If the dissonance is ignored and only the result of the second laboratory is considered, as was done during the trial, then the value of evidence amounts to 28 (or 1.45 in weight), which corresponds to moderate evidential support for the prosecution’s proposition. Thus, consideration of only the second test result has changed weak support for the prosecution’s proposition to moderate support.

**Table 5:** Summary of the probability values of the laboratory results for the prosecution and defense propositions $a_0$ and ${\bar{a}}_0$, respectively, extracted from the model of the Taffio Palmi - Busetto case with the corresponding likelihood ratio and weight values. The items of evidence are the negative result for the presumptive test ($d_{1}$), the negative DNA profile from the first laboratory ($d_{2}$) and the DNA profile from the second laboratory ($d_{3}$)

	$a_0$	${\bar{a}}_0$	Likelihood ratio	Weight
$Pr(d_1 \mid A_0)$	0.15	1.00	0.15	-0.82
$Pr(d_2 \mid d_1, A_0)$	0.59	1.00	0.59	-0.23
$Pr(d_3 \mid d_1, d_2, A_0)$	0.13	0.01	13.0	1.11
$Pr(d_3 \mid A_0)$	0.28	0.01	28.0	1.45

As with the interaction measure, only the conditioning proposition (here, $A_0$) must have two possible responses, while this requirement does not extend to descendant nodes, provided that the relative sizes of the weights involved in computing the dissonance measure can be determined as entailed by Eq. 18. It is also important to note that applying a dissonance analysis using the proposed method requires the weights to be specified, but not the exact reasoning structure itself.

These characteristics, much like the interaction measure discussed earlier, lends itself to broader applicability, as it allows for the examination of model properties without requiring deep expertise in the underlying reasoning framework proposed in Sect. 2. Consequently, this makes the approach particularly useful for non-experts in modeling, enabling them to assess the overall coherence of the assumption underlying the model by focusing on the impact of different weight assignments rather than intricate structural details.

## Conclusion

The importance of understanding evidential phenomena occurring in combined evidence is well known to practitioners of law, who are involved in evidence-based reasoning on a daily basis (Wigmore (1937), e.g.), scientists and statisticians, who are engaged in the subject of evidence interpretation (see for example Lindley’s Foreword in (Aitken et al. 2021, p. xxiv)). However, the scientific literature on these subjects remains sparse even today. In an attempt to advance the research in this field, the authors constructed and examined possible quantitative descriptions for evidential phenomena recurrently occurring in combined evidence and masses of evidence. The notion of Good’s weight of evidence is extremely useful in this endeavour mainly since the weight of evidence provides an additive metric (Good (1950, 1983), e.g.).

The case examples in Sects. 3.6 and 4.4 illustrate the help that can be given to case investigations and evidential evaluations by proper consideration of the evidential phenomena discussed here. In Sect. 3.6, Case 1 provides an example of redundance. Failure to consider this would increase the value of the evidence by a factor greater than six, as measured by the likelihood ratio. Case 2 provides an example of conditional independence indicated by a value of zero for the interaction measure. Case 3 provides an example of synergy that leads to an increase by nearly a factor of nine in the value of the evidence. The Taffio Palmi - Busetto case of Sect. 4.4 provides an example of dissonance such that the value of the evidence may be determined to be just a little over one in favour of the prosecution ($10^{0.06} = 1.15$, weak evidence) with a corresponding value of evidence lost to dissonance, compared to the potential value, of over 120. In contrast, the evidence relied on at the trial was only the second laboratory test which provided a value of nearly 30 (28.0), moderate support for the prosecution’s proposition.

Many of the phenomena discussed here have been first described by Schum (1994) from a probabilistic point of view. However, a general quantitative description of these phenomena did not exist and needed first to be devised. The present research extends Schum’s previous works so as to facilitate the analysis of combined evidence. The general formulation of the measures devised here can be applied recursively to sets and subsets of evidence similar to a scheme suggested in Schupbach (2011) for a measure of coherence introduced by Shogenji (1999). This feature enables one to analyse large bodies of evidence not only as a whole, but also down to their particular details. Such analyses could not be effectively realised up to now. Although the methods developed in this paper require only the weight of evidence for each item to be known, obtaining these weights typically involves complex reasoning models. Constructing such models can be challenging in practice, as they often require advanced analytical techniques and a high level of modeling expertise. The methods developed in this paper can also assist in constructing models that incorporate multiple items of evidence by enabling a more targeted cross-validation of modeling assumptions against the results generated by these methods. At present, the application of the interaction measure and the dissonance measure is limited to weights with two conditioning propositions. Future research is needed to extend these measures to cases where conditioning propositions have more than two possible responses. That said, these measures not only improve the understanding of combined evidence in case-based reasoning or model construction but also contribute to the investigation of combined evidence from a more general perspective. It may also be of interest to investigate the possible recognition by the human mind of the inferential interactions among items of evidence. The current work develops measures based on conditional probabilities (Strittmatter et al. (2024), e.g.) rather than a direct determination of the interaction. Perhaps systems and processes can be established to enable such a direct determination to be made. Perhaps there are certain conditions that enable a determination to be made. Identification of these conditions may then enable optimal exploitation of them in probabilistic systems. As has been shown, the presence of dissonant evidence can lead to a significant change in the value of the evidence, for example, through a change in the category of verbal support from ‘very strong’ to ‘weak’ support for a proposition. The calculation of any value for a likelihood ratio depends on assumptions and an associated model. The value of a likelihood ratio is the value conditional on the underlying model and assumptions. They are part of the background and should be included in the expert’s report. An expert can be challenged on the choice of model and the assumptions. If offered a different model or different assumptions, the expert can offer a new evaluation which may or may not be the same as the original one. Two experts may give different values for a particular piece of evidence because they have different models and assumptions. These different values may lead to support for opposing propositions. It is then a matter for the trier of fact (judge / jury) to decide with which model, assumptions and value they agree. Ideally, any differences between experts can be resolved in a pre-trial meeting but agreement may not always happen. The model and assumptions used should always be part of an expert’s report.

## Appendix A: Interpretation of likelihood ratio and weight of evidence values

Table 6 maps the value ranges of the likelihood ratio and weight of evidence to specific situations of evidential support. A likelihood ratio of say 100 means that the evidential support for the proposition in the numerator is 100 times stronger than for the proposition in the denominator. This equates to the evidence having a weight of 2 in favour of the proposition in the numerator over the proposition in the denominator, assuming that a base 10 logarithm is taken.

Likelihood ratio and weight of evidence values can also be expressed using a verbal scale. Verbal scales may be a valuable tool to convey numerical results to a non-scientific audience help non-scientists in understanding what is considered ‘weak’ or ‘strong’ evidence by forensic science professionals. Verbal scales are usually employed for likelihood ratio values larger than one (i.e. weights larger than zero). The verbal sale employed for likelihood ratios larger than one (or weights larger than zero) in Table 6 is taken from Marquis et al. (2016). Reciprocals of the likelihood ratio and negative values of weights provide corresponding evidential support for the proposition in denominator.

**Table 6:** Interpretation of likelihood ratio and weight of evidence values in terms of evidential support

Likelihood ratio (*L*)	Weight of evidence (*W*)	Evidential support
0	-∞	The evidence is impossible under the proposition in the numerator.
0<L<1	-∞<W<0	The evidence supports the proposition in the denominator over the proposition in the numerator.
L=1	W=0	The evidence supports the proposition in the denominator and numerator equally.
1<L	0<W	The evidence supports the proposition in the numerator over the proposition in the denominator.
1<L≤10	0<W≤1	Weak evidential support for the proposition in the numerator.
10<L≤100	1<W≤2	Moderate evidential support for the proposition in the numerator.
100<L≤1000	2<W≤3	Strong evidential support for the proposition in the numerator.
$1000<L\leq 10^{\prime }000$	3<W≤5	Very strong evidential support for the proposition in the numerator.
$L>10^{\prime }000$	W>5	Extremely strong evidential support for the proposition in the numerator.
∞	∞	The evidence is certain under the proposition in the numerator.

## Appendix B: Interpretation of $R_{d_2\mid d_1}$

Table 7 maps the different values of $R_{d_2\mid d_1}$ to the corresponding type of inferential interaction as given by Schum (1994).

**Table 7:** Relationship between the measure $R_{d_2\mid d_1}$ and the inferential force of $d_2$

$R_{d_2\mid d_1}$	$d_2$ supports $a_0$ over ${\bar{a}}_0$ ($W_{d_2\mid a_0} > 0$)	$d_2$ supports ${\bar{a}}_0$ over $a_0$ ($W_{d_2\mid a_0}<0$)	Type of interaction
$R_{d_2\mid d_1}<0$	$W_{d_2\mid d_1, a_0} > W_{d_2\mid a_0}$	$W_{d_2\mid d_1, a_0}<W_{d_2\mid a_0}$	Synergy
$R_{d_2\mid d_1} = 0$	$W_{d_2\mid d_1, a_0} = W_{d_2\mid a_0}$	$W_{d_2\mid d_1, a_0} = W_{d_2\mid a_0}$	Cond. independence
$1> R_{d_2\mid d_1} > 0$	$W_{d_2\mid a_0}> W_{d_2\mid d_1, a_0} > 0$	$W_{d_2\mid a_0}< W_{d_2\mid d_1, a_0} < 0$	$\Big \}$ Redundance
$R_{d_2\mid d_1} = 1$	$W_{d_2\mid d_1, a_0} = 0$	$W_{d_2\mid d_1, a_0} = 0$	
$R_{d_2\mid d_1} > 1$	$W_{d_2\mid d_1, a_0}<0$	$W_{d_2\mid d_1, a_0} > 0$	Directional change

## Appendix C: Inferential interaction in terms of $W_{d_1, d_2 \mid a_0}$

Table 8 maps definitions of inferential interactions to its implications on $W_{d_1, d_2 \mid a_0}$ for cases where $W_{d_1 \mid a_0} > 0$ and $W_{d_2\mid a_0} > 0$ (see Appendix D for the derivations of these relationships from (5)). For a more thorough discussion on the definitions of the types of inferential interactions see Schum (1994).

**Table 8:** Types of inferential interactions in terms of weight of evidence

Definition of inferential interaction	Inferential interaction in terms of $W_{d_1, d_2 \mid a_0}$
Synergy	
$W_{d_2\mid d_1, a_0} > W_{d_2\mid a_0}$	$W_{d_1, d_2 \mid a_0} > W_{d_1 \mid a_0} + W_{d_2\mid a_0}$
Conditional independence	
$W_{d_2\mid d_1, a_0} = W_{d_2\mid a_0}$	$W_{d_1, d_2 \mid a_0} = W_{d_1 \mid a_0} + W_{d_2\mid a_0}$
Partial redundance	
$0<W_{d_2\mid d_1, a_0} < W_{d_2\mid a_0}$	$W_{d_1 \mid a_0}< W_{d_1, d_2 \mid a_0} < W_{d_1 \mid a_0} + W_{d_2\mid a_0}$
Complete redundance	
$W_{d_2\mid d_1, a_0} = 0$	$W_{d_1, d_2 \mid a_0} = W_{d_1 \mid a_0}$
Directional change	
$W_{d_2\mid d_1, a_0}<0$	$W_{d_1, d_2 \mid a_0} < W_{d_1 \mid a_0}$

## Appendix D: Derivations for types of inferential interaction in terms of weight of evidence

In the case where both items of evidence provide weight in favour of $a_0$ ($W_{d_1 \mid a_0} > 0$ and $W_{d_2 \mid a_0} > 0$), the following derivations apply:

**Synergy** The events $d_1$ and $d_2$ are said to be inferentially synergistic given proposition $A_0$ if the inequality $W_{d_2 \mid d_1, a_0} > W_{d_2 \mid a_0}$ holds. A reformulation of this inequality into $0 > W_{d_2 \mid a_0} - W_{d_2 \mid d_1, a_0}$, and by applying (5) leads to the conclusion that the joint weight of evidence of $d_1$ and $d_2$ is larger than the sum of each weight, that is, $W_{d_1, d_2 \mid a_0} > W_{d_1 \mid a_0} + W_{d_2 \mid a_0}$.

**Conditional independence** The events $d_1$ and $d_2$ are said to be conditionally independent given proposition $A_0$ if $Pr(d_2 \mid d_1, A_0) = Pr(d_2 \mid A_0)$. In other words, one has $W_{d_2 \mid d_1, a_0} = W_{d_2 \mid a_0}$. Hence, $W_{d_2\mid a_0} - W_{d_2\mid d_1, a_0} = 0$, and from (5) it follows that $W_{d_1, d_2 \mid a_0} = W_{d_1 \mid a_0} + W_{d_2 \mid a_0}$.

**Partial redundance** An event $d_2$ is said to be **partially** redundant given $d_1$, if $0< W_{d_2\mid d_1, a_0} < W_{d_2 \mid a_0}$ holds. Hence, (5) states that the lower boundary of the joint weight of evidence in favour of *h* provided by $d_2$ and $d_1$ is $W_{d_1 \mid a_0} < W_{d_1, d_2 \mid a_0}$, and that the upper boundary is $W_{d_1, d_2 \mid a_0} < W_{d_1 \mid a_0} + W_{d_2 \mid a_0}$ (i.e., $W_{d_1 \mid a_0}< W_{d_1, d_2 \mid a_0} < W_{d_1 \mid a_0} + W_{d_2 \mid a_0}$). In other words, the domain of values associated with redundance is bounded by the values of complete redundance ($W_{d_1, d_2 \mid a_0} = W_{d_1 \mid a_0}$) and conditional dependence ($W_{d_1, d_2 \mid a_0} = W_{d_1 \mid a_0} + W_{d_2 \mid a_0}$).

**Complete redundance** Event $d_2$ is said to be completely redundant given $d_1$ and proposition $A_0$, if $W_{d_2 \mid d_1, a_0} = 0$ holds. In such cases (5) reduces to $W_{d_1, d_2 \mid a_0} = W_{d_1 \mid a_0}$.

**Directional change** The occurrence of event $d_1$ is said to induce a directional change in $d_2$ given proposition $A_0$ (i.e., event $d_2$ supports ${\bar{a}}_0$ once $d_1$ is known to have occurred), if $W_{d_2 \mid d_1, a_0} < 0$ holds. From (5) it follows that $W_{d_1, d_2 \mid a_0} < W_{d_1 \mid a_0}$. The derivations for cases where $W_{d_1 \mid a_0} < 0$ and $W_{d_2 \mid a_0} < 0$ can be obtained analogously.

## Appendix E: Values of $IA_{{\textbf{D}}_k \mid a_0}$ and their implication on the presence of a directional change or synergy

### E.1: Proving that $IA_{{\textbf{D}}_k \mid a_0} > 1$ implies $W_{{\textbf{D}}_{dc} \mid a_0} \ne 0$

The statement that in all cases where $W_{d_{i, j} \mid a0} > 0, d_{i,j} \in {\textbf{D}}_k$, an interaction value larger than one implies the presence of an effect of directional change ($W_{{\textbf{D}}_{dc}\mid a_0} \ne 0$) is to be proven here. The proof is established by exploiting the fact that a value of $IA_{{\textbf{D}}_k \mid a_0} > 1$ being equivalent to $W_{{\textbf{D}}_{k} \mid M, a_0} > 0$ and $W_{{\textbf{D}}_{k} \mid a_0} < 0$, implies that $W_{{\textbf{D}}_{dc}\mid a_0} \ne 0$. More formally we have,

20 $$\begin{aligned}&IA_{{\textbf{D}}_k \mid a_0}> 1&\Longleftrightarrow W_{{\textbf{D}}_{k} \mid M, a_0} > 0 \text { and } W_{{\textbf{D}}_{k} \mid a_0} < 0 \text {, and} \end{aligned}$$

21 $$\begin{aligned}&W_{{\textbf{D}}_{k} \mid M, a_0} > 0 \text { and } W_{{\textbf{D}}_{k} \mid a_0} < 0&\Longrightarrow W_{{\textbf{D}}_{dc}\mid a_0}\ne 0 . \end{aligned}$$

The equivalence stated in (20) follows directly from (8). This equation is used again as a starting point for the demonstration of statement (21), notably

$$\begin{aligned} IA_{{\textbf{D}}_k \mid a_0} = \frac{W_{{\textbf{D}}_k \mid M, a_0} - W_{{\textbf{D}}_k \mid a_0}}{W_{{\textbf{D}}_k \mid M, a_0}} > 1, \end{aligned}$$

from where it follows that

$$\begin{aligned} -W_{{\textbf{D}}_{k} \mid a_0}&> 0 \\ -W_{{\textbf{D}}_{s}\mid a_0} - W_{{\textbf{D}}_{pr}\mid a_0} - W_{{\textbf{D}}_{dc}\mid a_0}&> 0.\\ \end{aligned}$$

Finally, one obtains

$$\begin{aligned} - W_{{\textbf{D}}_{dc}\mid a_0} > W_{{\textbf{D}}_{s}\mid a_0} + W_{{\textbf{D}}_{pr}\mid a_0}. \end{aligned}$$

For this inequality to hold, it is necessary that $W_{{\textbf{D}}_{dc} \mid a_0} \ne 0$ since $W_{{\textbf{D}}_{s}\mid a_0} \ge 0$, $W_{{\textbf{D}}_{pr} \mid a_0} \ge 0$, and $W_{{\textbf{D}}_{dc} \mid a_0} <0$ by definition (see D) for cases in which $W_{d_{i,j} \mid M, a_{0}} > 0$ for all $d_{i,j} \in {\textbf{D}}_{k}$; that is, each item of evidence considered in isolation points to the same proposition of interest. This result allows (20) and (21) to be rewritten in a more informative manner

$$\begin{aligned} IA_{{\textbf{D}}_k \mid a_0}> 1 \Longleftrightarrow - W_{{\textbf{D}}_{dc}\mid a_0}&> W_{{\textbf{D}}_{s}\mid a_0} + W_{{\textbf{D}}_{pr}\mid a_0} \Longrightarrow W_{{\textbf{D}}_{dc}\mid a_0}\ne 0. \end{aligned}$$

By applying the same reasoning to situations, in which $W_{d_{i, j} \mid a0} < 0, d_{i,j} \in {\textbf{D}}_k$ one obtains

$$\begin{aligned} IA_{{\textbf{D}}_k \mid a_0}< 1 \Longleftrightarrow - W_{{\textbf{D}}_{dc}\mid a_0}&< W_{{\textbf{D}}_{s}\mid a_0} + W_{{\textbf{D}}_{pr} \mid a_0} \Longrightarrow W_{{\textbf{D}}_{dc} \mid a_0}\ne 0. \end{aligned}$$

### E.2: Proving that $IA_{{\textbf{D}}_k \mid a_0} < 0$ implies $W_{{\textbf{D}}_{s}\mid a_0}\ne 0$

In situations, in which $W_{d_{i, j} \mid a0} > 0$ where $d_{i,j} \in {\textbf{D}}_k$ a value of $IA_{{\textbf{D}}_k \mid a_0}<0$ indicates that ${\textbf{D}}_{s} \ne 0$ ($W_{D_{s} \mid a_{0}} \ne 0$). More precisely, one has the relationships

22 $$\begin{aligned}&IA_{{\textbf{D}}_k \mid a_0}<0&\Longleftrightarrow W_{{\textbf{D}}_{k} \mid M, a_0} > W_{{\textbf{D}}_{k} \mid a_0} \text {, and} \end{aligned}$$

23 $$\begin{aligned}&W_{{\textbf{D}}_{k} \mid M, a_0} > W_{{\textbf{D}}_{k} \mid a_0} \Longrightarrow W_{{\textbf{D}}_{s}\mid a_0}\ne 0. \end{aligned}$$

The equivalence in (22) follows directly from (8), which is used − just as in E.1 − to prove the implication in (23). Notably,

$$\begin{aligned} IA_{{\textbf{D}}_k \mid a_0} = \frac{W_{{\textbf{D}}_k \mid M, a_0} - W_{{\textbf{D}}_{k} \mid a_0}}{W_{{\textbf{D}}_k \mid M, a_0}} < 0, \end{aligned}$$

where $$W_{{\textbf{D}}_k \mid M, a_0} \ne 0$$. Thus,

$$\begin{gathered} W_{{\left. {{\mathbf{D}}_{k} } \right|a_{0} }} > W_{{\left. {{\mathbf{D}}_{k} } \right|M,a_{0} }} \hfill \\ W_{{\left. {{\mathbf{D}}_{s} } \right|a_{0} }} + W_{{\left| {\left. {{\mathbf{D}}_{{pr}} } \right|a_{0} } \right.}} + W_{{\left. {{\mathbf{D}}_{{dc}} } \right|a_{0} }} > W_{{\left. {{\mathbf{D}}_{k} } \right|M,a_{0} }} , \hfill \\ \end{gathered}$$

and finally one has

$$\begin{aligned} W_{{\textbf{D}}_{s}\mid a_0} > W_{{\textbf{D}}_k \mid M, a_0} -W_{{\textbf{D}}_{pr}\mid a_0} - W_{{\textbf{D}}_{dc}\mid a_0}. \end{aligned}$$

For this inequality to hold, it is necessary that an effect of synergy is present ($W_{{\textbf{D}}_{s}\mid a_0} \ne 0$) since $W_{{\textbf{D}}_k \mid M, a_0}$, and $W_{{\textbf{D}}_{pr}\mid a_0}$ take values larger than zero each and $W_{{\textbf{D}}_{dc}\mid a_0}$ a value smaller than zero by definition (see Appendix D). Thus one can write

$$\begin{aligned} IA_{{\textbf{D}}_k \mid a_0} < 0 \Longleftrightarrow W_{{\textbf{D}}_{s}\mid a_0} > W_{{\textbf{D}}_k \mid M, a_0} -W_{{\textbf{D}}_{pr}\mid a_0} - W_{{\textbf{D}}_{dc}\mid a_0} \Longrightarrow W_{{\textbf{D}}_{s}\mid a_0}\ne 0. \end{aligned}$$

Applying an analogous reasoning to situations, in which $W_{d_{i, j} \mid a0} < 0$ where $d_{i,j} \in {\textbf{D}}_k$ leads to

$$\begin{aligned} IA_{{\textbf{D}}_k \mid a_0} > 0 \Longleftrightarrow W_{{\textbf{D}}_{s}\mid a_0} < W_{{\textbf{D}}_k \mid M, a_0} -W_{{\textbf{D}}_{pr}\mid a_0} - W_{{\textbf{D}}_{dc}\mid a_0} \Longrightarrow W_{{\textbf{D}}_{s}\mid a_0}\ne 0. \end{aligned}$$

## Appendix F: Derivation of values of $W^\text {diss}_{{\textbf{D}} \mid a_0}$

Let ${\textbf{D}}_k$ denote the subset of items providing positive weight and the subset ${\textbf{D}}_{n - k}$ the subset of items providing negative weight. Equation (18) can be written as

24 $$\begin{aligned} \begin{aligned} W^\text {diss}_{{\textbf{D}} \mid a_0} = \overbrace{\left| W_{{\textbf{D}}_{k} \mid a_0}\right| + \left| W_{{\textbf{D}}_{n - k} \mid a_0} \right| }^{\Vert W_{{\textbf{D}} \mid a_0}\Vert } - \overbrace{\left| W_{{\textbf{D}}_{k} \mid a_0} + W_{{\textbf{D}}_{n - k} \mid a_0} \right| }^{\left| W_{{\textbf{D}} \mid a_0}\right| } \\ = W_{{\textbf{D}}_{k} \mid a_0} - W_{{\textbf{D}}_{n - k} \mid a_0} - \left| W_{{\textbf{D}}_{k} \mid a_0} + W_{{\textbf{D}}_{n - k} \mid a_0} \right| . \end{aligned} \end{aligned}$$

A case-by-case analysis of (24) produces (19): *Case (i).* If $W_{{\textbf{D}}_{k} \mid a_0} < -W_{{\textbf{D}}_{n - k} \mid a_0}$, (24) becomes

$$\begin{aligned} W^\text {diss}_{{\textbf{D}} \mid a_0} = W_{{\textbf{D}}_{k} \mid a_0} - W_{{\textbf{D}}_{n - k} \mid a_0} - \left( -W_{{\textbf{D}}_{k} \mid a_0} - W_{{\textbf{D}}_{n - k} \mid a_0} \right) = 2W_{{\textbf{D}}_{k} \mid a_0}. \end{aligned}$$

*Case (ii).* If $W_{{\textbf{D}}_{k} \mid a_0} = -W_{{\textbf{D}}_{n - k} \mid a_0}$ and (24) becomes

$$\begin{aligned} W^\text {diss}_{{\textbf{D}} \mid a_0} = \Vert W_{{\textbf{D}} \mid a_0}\Vert = W_{{\textbf{D}}_{k} \mid a_0} - W_{{\textbf{D}}_{n - k} \mid a_0} , \end{aligned}$$

which is equivalent to $2W_{{\textbf{D}}_{k} \mid a_0}$ and $-2W_{{\textbf{D}}_{n - k} \mid a_0}$ respectively.

*Case (iii).* If $W_{{\textbf{D}}_{k} \mid a_0} > -W_{{\textbf{D}}_{n - k} \mid a_0}$ (24) becomes

$$\begin{aligned} W^\text {diss}_{{\textbf{D}} \mid a_0} = W_{{\textbf{D}}_{k} \mid a_0} - W_{{\textbf{D}}_{n - k} \mid a_0} - \left( W_{{\textbf{D}}_{k} \mid a_0} + W_{{\textbf{D}}_{n - k} \mid a_0} \right) = -2W_{{\textbf{D}}_{n - k} \mid a_0} . \end{aligned}$$

## Footnotes

1 The word ‘mass’ was first introduced by Wigmore (1937). Schum (1994) discusses the idea of a ‘mass of evidence’, where a ‘mass’ may be thought of as multiple items of evidence. The question of interest is how evidential reasoning may be based on knowledge of these multiple items.

2 In what follows, the terms *report* and *evidence* are used synonymously and denoted *r* (or *R*, depending on the context). The event about which the report is made is denoted *E*.

3 Consider the following scenario to illustrate this concept. You wake up at the night to fetch a glass of water in the kitchen. In the hallway you turn on the light, but it fails to illuminate. You hypothesise that either the light bulb is broken or there is a power outage. Initially, the weight you assign to the failed illumination favours the first proposition over the power outage. If you had also tried the bedroom light, would its status affect the weight you currently hold? If your answer is yes, then you view the illumination status in the bedroom and hallway as conditionally dependent given your propositions. Otherwise, you consider them conditionally independent.

4 This measure of inferential interaction refers to Schum’s descriptions of inferential interaction and is not to be confused with the interactions of weight of evidence as outlined by Good (1960).

5 The subscript *i* in $j_{i}$ is omitted if the context renders it unnecessary.

6 Note the change of notation. Items of evidence are from now on denoted *D* for descendant nodes rather than *R* for report. Events about which *D* is evidence are now denoted *A* for ancestor rather than *E* for event.

7 The notation *R* here for redundance measure is not to be confused with the use of *R* for report in Sect. 1.1

8 The term ‘straw model’ is introduced following Laskey (1991). There it is suggested that the use of the ‘straw model’, where the items are independent given $a_0$, is essentially a reference model of a particular choice. In other situations one may see a better choice in using a reference model capturing an expert’s opinion.

9 In particular, the computation is based on the version of the model that includes the contamination variable and employs the original parameters discussed in the paper and provided in the supplemental material. Moreover, the probability of contamination was set to 1%. The variables for the DNA sampling method on the necklace for the first and second laboratory were set (instantiated) to ‘swap’ and ‘immersion’ respectively. The probabilities of the proposition for the prosecution and defence are set to be equal. The Hugin Researcher 9.2 software was used for the computations.

## References

[CR1] Aitken C, Nordgaard A, Taroni F, Biedermann A (2018) Comementary: likelihood ratio as weight of forensic evidence. Front Genet 9:224–225 10.3389/fgene.2018.00224 PMC6036285 30013593

[CR2] Aitken CGG, Taroni F, Bozza S (2021) Statistics and the evaluation of evidence for forensic scientists. John Wiley & Sons, Chichester

[CR3] Bernardo JM, Smith AFM (2000) Bayesian theory. John Wiley & Sons, Chichester

[CR4] Crupi V, Tentori K (2016) Confirmation theory. In: Ajek AH, Hitchock C (eds) Oxford handbook of philosophy and probability. Oxford University Press, Oxford, pp 650–665

[CR5] Crupi V, Tentori K, Gonzalez M (2007) On Bayesian measures of evidential support: theoretical and empirical issues. Philos Sci 74(2):229–252

[CR6] Darwiche A (2009) Modeling and reasoning with Bayesian networks. Cambridge University Press, New York

[CR7] de Finetti B (2010) Philosophical lecture on probability - collected, edited, and annotated by Alberto Mura. Synthese Library. Sassari: Springer Science+Business Media B.V

[CR8] De March I, Taroni F (2020) Bayesian networks and dissonant items of evidence: a case study. Forensic Sci Int Genet 44:102172 10.1016/j.fsigen.2019.102172 31629186

[CR9] Garrett AJM (1998) The history of probability theory. In: Erickson G, Rychert J, Smith C (Eds.), Maximum entropy and Bayesian methods, vol 98. Fundamental theories of physics. Netherlands, Springer, pp 223–238

[CR10] Glass DH, McCartney M (2015) A new argument for the likelihood ratio measure of confirmation. Acta Anal 30:59–65

[CR11] Gittelson S, Berger CEH, Jackson G, Evett IW, Champod C, Robertson B, Curran JM, Taylor D, Weir BS, Coble MD, Buckleton JS (2018) A new argument for the likelihood ratio measure of confirmation. Forensic Sci Int 288:15–19 10.1016/j.forsciint.2018.05.025 PMC7306225 29857959

[CR12] Good IJ (1950) Probability and the weighing of evidence. Butler and Tanner Ltd, London

[CR13] Good IJ (1960) Effective sampling rates for signal detection; or can the Gaussian model be salvaged?. Information and control, 3(116-140)

[CR14] Good IJ (1983) Good thinking - the foundations of probability and its applications. Dover Publications Inc, Mineola

[CR15] Good IJ, Card WI (1971) The diagnostic process with special reference to errors. Method Inf Med 10:176–188 5571982

[CR16] Jensen FV, Nielsen TD (2007) Bayesian networks and decision graphs. Springer, New York

[CR17] Juchli P, Biedermann A, Taroni F (2012) Graphical probabilistic analysis of the combination of items of evidence. Law Probab Risk 11:51–84

[CR18] Kjaerulff UB, Madsen AL (2013) Bayesian networks and influence diagrams: a guide to construction and analysis. Springer, New York, NY

[CR19] Laskey KB (1991) Conflict and surprise: heuristics for model revision. In: Uncertainty proceedings 1991. Elsevier, pp 197–204

[CR20] Lindley DV (2006) Understanding uncertainty. John Wiley & Sons, Hoboken

[CR21] Lund SP, Iyer H (2017) Likelihood ratio as weight of forensic evidence: a closer look. J Res Nat Inst Stand Technol 122:1–32 10.6028/jres.122.027 PMC7339646 34877093

[CR22] Marquis R, Biedermann A, Cadola L, Champod C, Gueissaz L, Massonnet G, Mazzella WD, Taroni F, Hicks T (2016) Discussion on how to implement a verbal scale in a forensic laboratory: benefits, pitfalls and suggestions to avoid misunderstandings. Sci Justice 56:364–370 10.1016/j.scijus.2016.05.009 27702452

[CR23] Pearl J (1988) Probabilistic reasoning in intelligent systems: networks of plausible inference. Morgan Kaufmann Publishers Inc, San Mateo, CA

[CR24] Schum DA (1981) Sorting out the effects of witness sensitivity an response-criterion placement upon the inferential value of testimonial evidence. Organ Behav Hum Perform 27:153–196

[CR25] Schum DA (1994) The evidential foundations of probabilistic reasoning. John Wiley & Sons, New York

[CR26] Schum DA, Martin AW (1982) Formal and empirical research on cascaded inference in jurisprudence. Law Soc Rev 1:105–152

[CR27] Schupbach JN (2011) New hope for Shogenji’s coherence measure. The British journal for the philosophy of science

[CR28] Shogenji T (1999) Is coherence truth conducive? Analysis 59(4):338–345

[CR29] Strittmatter LE, Pilditch TD, Lagnado D (2024) Reasoning about (in)dependent evidence: a mismatch between perceiving and incorporating dependencies? In: Samuelson LK, Frank SL, Toneva M, Mackey A, and Hazeltine E (Eds.), Proceedings of the 46th annual conference of the cognitivescience society

[CR30] Taroni F, Aitken CGG, Bozza S, Juchli P (2024) The principle of total evidence reprised. Law Probab Risk 23:1–23

[CR31] Taroni F, Biedermann A, Garbolino P, Aitken CGG (2004) A general approach to Bayesian networks for the interpretation of evidence. Forensic Sci Int 139:5–16 10.1016/j.forsciint.2003.08.004 14687767

[CR32] Taroni F, Juchli P, Aitken CGG (2020) A probabilistic account of the concept of cross-transfer and inferential interactions for trace material. Law Probab Risk 19:221–233

[CR33] Taroni F, Garbolino P, Bozza S, Aitken CGG (2021) Bayes’ factor: the coherent measure for hypothesis confirmation. Law Probab Risk 20:15–36

[CR34] Thompson WC, Taroni F, Aitken CGG (2003) How the probability of a false positive affects the value of DNA evidence. J Forensic Sci 48:47–54 12570198

[CR35] Tillers P (1989) Webs of things in the mind: a new science of evidence. Mich Law Rev 87:1225–1258

[CR36] Wigmore JH (1937) The science of judicial proof: as given by logic, psychology, and general experience and illustrated in judicial trials, 3rd edn. Brown, Boston

